# Reverse Chemical Genetics: Comprehensive Fitness Profiling Reveals the Spectrum of Drug Target Interactions

**DOI:** 10.1371/journal.pgen.1006275

**Published:** 2016-09-02

**Authors:** Lai H. Wong, Sunita Sinha, Julien R. Bergeron, Joseph C. Mellor, Guri Giaever, Patrick Flaherty, Corey Nislow

**Affiliations:** 1 Department of Pharmaceutical Sciences, University of British Columbia, Vancouver, Canada; 2 Department of Biochemistry, University of Washington, Seattle, Washington, United States of America; 3 seqWell, Inc., Beverly, Massachusetts, United States of America; 4 Department of Mathematics and Statistics, University of Massachusetts, Amherst, Massachusetts, United States of America; Stanford University School of Medicine, UNITED STATES

## Abstract

The emergence and prevalence of drug resistance demands streamlined strategies to identify drug resistant variants in a fast, systematic and cost-effective way. Methods commonly used to understand and predict drug resistance rely on limited clinical studies from patients who are refractory to drugs or on laborious evolution experiments with poor coverage of the gene variants. Here, we report an integrative functional variomics methodology combining deep sequencing and a Bayesian statistical model to provide a comprehensive list of drug resistance alleles from complex variant populations. Dihydrofolate reductase, the target of methotrexate chemotherapy drug, was used as a model to identify functional mutant alleles correlated with methotrexate resistance. This systematic approach identified previously reported resistance mutations, as well as novel point mutations that were validated *in vivo*. Use of this systematic strategy as a routine diagnostics tool widens the scope of successful drug research and development.

## Author Summary

One of the most profound outcomes of fast, reliable genome sequencing is the ability to tailor drug therapy to an individual’s genotype. This ‘personalized’ or ‘precision medicine’ is the realization of a decades-long effort to maximize drug effect and limit unwanted side effects. An undesirable consequence of such targeted therapies, however, is the emergence of drug resistance. This outcome is the result of an evolutionary process where mutations in the drug target render the drug perturbation allow such mutant cells to proliferate. Because of the unbiased, and stochastic nature of the emergence of drug resistance, it is impossible to predict. We developed a test where hundreds of thousands of mutant cells are exposed to a drug simultaneously and those cells that modulate resistance survive. This method is innovative because it partners a high-throughput experimental protocol with a tailored statistical model to identify all mutations that modulate resistance. Finally, we used synthetic biology to re-create these mutations and demonstrate that they were, in fact, bona fide drug-resistant variants. These mutations were further extended and confirmed to also be resistant in the human orthologue. This combined biological-computational approach allows one to identify drug’s degree of resistance to both guide treatments and future drug discovery.

## Introduction

Drug resistance is a worldwide health concern that affects all drug classes, including anti-infectives and anti-cancer agents [1–3]. Recent reports illustrate that first-line antibiotic treatment failure rates have increased 12% from 1991–2012 [4]. Cancer drug resistance has increased, in part due to the use of highly specific targeted therapeutics [1,5]. While attempts to combine drugs into “smart cocktails” hold some promise to reduce emergence of resistance, in the majority of cases drug resistance is inevitable. Therefore, it is important to understand the causative mechanisms of resistance to improve the use and targeting of therapeutics.

Current strategies for understanding the mechanisms of resistance include: i) observational trials [1,6], ii) *in situ* mutagenesis [7–10] and iii) computational approaches [7,11–13]. However, each of these methods suffer from limitations with respect to throughput, resolution and accuracy. Hence, a rapid, systematic and cost-effective strategy to identify gene variants that modulate drug resistance over time is required to improve our understanding of resistance mechanisms.

Here, we present such a streamlined method to identify the emergence and persistence of modulators of drug resistance. Our integrative approach combines a strategic parallel competitive *in vivo* resistance assay with a Bayesian statistical model [14,15] that is both systematic and quantitative. We applied this assay to the anti-cancer drug methotrexate (MTX) in its well-characterized target, dihydrofolate reductase. Our pipeline takes advantage of the *S*. *cerevisiae* variomics collection, which contains libraries of 2 x 10^5^ random plasmid-borne point mutation alleles for every yeast gene [16]. These alleles are packaged within haploid-convertible heterozygous diploid yeast gene knockouts which can be grown competitively and quantified with massively parallel sequencing.

Yeast dihydrofolate reductase (*DFR1*) is a validated functional orthologue of human dihydrofolate reductase (hDHFR), which is commonly used to study the MTX mechanism of action and enzymology [15,17,18]. In previous work, yeast has been employed to study genome-wide gene-drug interactions [19–23], and is a well-established model for anticancer drug research [6,17,24,25]. Methotrexate acts as an antimetabolite that targets the enzyme dihydrofolate reductase, which functions to maintain folate homeostasis in nucleus and mitochondria by reducing dihydrofolate into tetrahydrofolate as a key element of thymidylate and protein synthesis [15]. Due to the high degree of conservation between yeast and human cellular pathways, the results obtained for the yeast dihydrofolate reductase can provide insights into how tumors acquire drug resistance, which is a major barrier to effective cancer treatment [26–28] and point mutations in DHFR active site have been shown to affect MTX binding affinity altering in turn MTX efficacy [8–10,29–37]. Thus, systematically surveying the causative *DFR1* point mutations that correlate with poor MTX response and understanding how resistant *dfr1* alleles interact with MTX will help develop MTX analogues with a potentially lower likelihood of resistance.

## Results

We first describe our novel integrative experimental and statistical analysis method. We then apply this method to the identification of variants that modulate resistance to methotrexate in its target, dihydrofolate reductase. We next present validation studies using reconstituted individual mutations grown in isolation. Finally, we use a DFR1 protein model to provide structure/function relationship analysis of the validated mutations.

### Parallel *in vivo* screening combined with parallel sequencing

**Fig 1:**
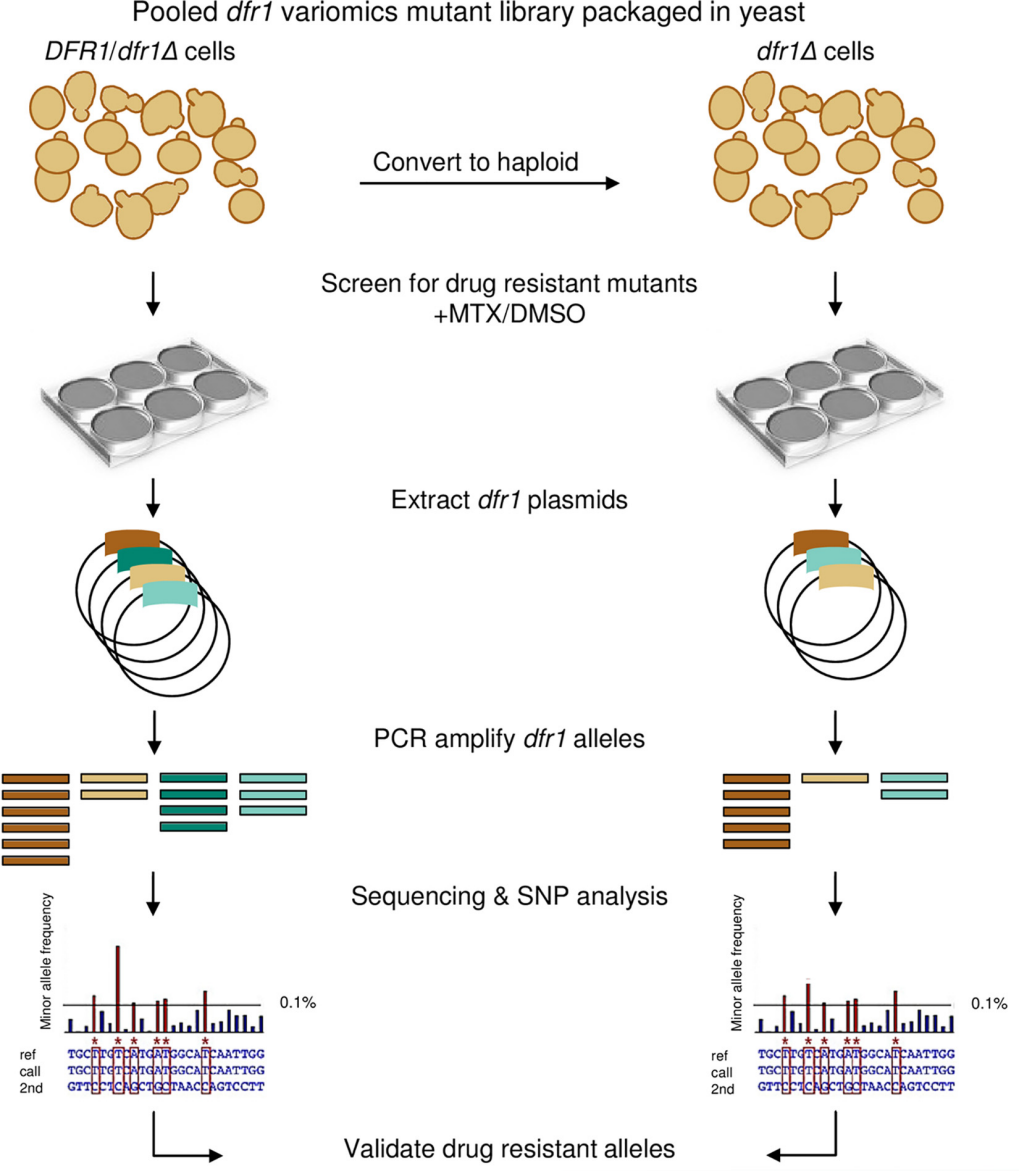
Workflow for methotrexate resistance screen. The plasmid-borne *dfr1* variomics library contains ~2 x 10^5^ independent *dfr1* alleles maintained in a diploid *DFR1*/*dfr1*Δ heterozygote strain. The diploid pool was sporulated and cultured in haploid selective media to generate a *dfr1* pool in a *dfr1*Δ haploid background. Both diploid and haploid strains were grown in the presence of MTX (2 mM) or DMSO solvent control (2% v/v) over a 6-day timecourse (details in S2 Fig). MTX-treated cultures were subsequently harvested for plasmid extraction followed by PCR amplification of the dfr1 alleles. Nextera XT libraries were prepared for DFR1-amplicon sequencing, and non-reference *dfr1* variant alleles were identified using the RVD analysis tool. Candidate *dfr1* point mutations were validated by constructing the individual *dfr1* mutants and their MTX resistance confirmed in growth assays.

The functional variomics technology was adapted in our study by using the original *dfr1* variomics library, which contains 2 x 10^5^ point mutations in *DFR1* [16]. To recover as many distinct *dfr1* MTX resistant-alleles as possible, we exploited the variomics tool by screening the diploid and haploid *dfr1* pools using an improved screening assay (Fig 1 and Methods). Specifically, we wanted to test if the resulting alleles differed depending on if the wild-type *DFR1* allele was present, as is the case for the *DFR1*/*dfr1*Δ heterozygote strain, or absent as in the haploid *dfr1*Δ strain. For haploids, the *dfr1* allele must maintain viability and provide drug resistance whereas in the diploid case, the wild type allele can in principle allow separation-of-function alleles (i.e. resistance without viability) to be recovered.

We tuned the parameters of the drug resistance assay to maximize for the enrichment of *dfr1* alleles in parallel competitive conditions in an attempt to mimic the environment in which heterogeneous tumors are exposed to cytostatic drugs [38,39] (Fig 1). The *dfr1* diploid library was first grown without drug selection to generate a *dfr1* pool with ~50-fold coverage per variant for each of the 2 x 10^5^ independent variants (see Methods for details). The pool was then induced to sporulate to generate a haploid *dfr1* pool of 2.2 x 10^4^ viable *dfr1* alleles which were then challenged with drug in liquid media. To minimize the loss of rare *dfr1* alleles, drug exposure was limited to a 6-day treatment of the diploid and haploid pools in liquid media at a MEC_100_ dose of MTX (Fig 1 and S1 Fig). Treated samples were collected every 2 days (equivalent to 8 generations of growth) and the remaining *dfr1* pools were further propagated in fresh media with MTX (S2 Fig). MTX-treated pools were harvested at each time point and plasmid-borne *dfr1* alleles were PCR amplified and sequenced at a median coverage of 10K (Fig 1 and S1 Table; Methods).

### Rare Variant Detection (RVD) analysis method to identify *dfr1* variants that modulate resistance to methotrexate

The sequencing data was collected in separate runs for the diploid and haploid experiments and each processed independently (see Methods for details). To call variants and estimate their associated allele frequencies in the mixed *dfr1* pools, we used our previously published rare variant detection statistical model (RVD2) [14]. We estimated the parameters of the model for each time point and for the wild-type control using the default Gibbs sampling and Markov chain monte carlo parameters (4000 Gibbs samples, 10 Metropolis-Hastings samples per gibbs sample, 20% warm-up, thinning rate of 2). Finally, we called variants using the somatic test function in RVD2. This test identifies variants where the difference in the non-reference read rate is between 0.1% and 100% between a designated case and control sample (95% posterior confidence). This test also filters for variant loci that have non-uniform, non-reference read counts to eliminate false-positive calls due to generally elevated sequencing error rates.

**Fig 2:**
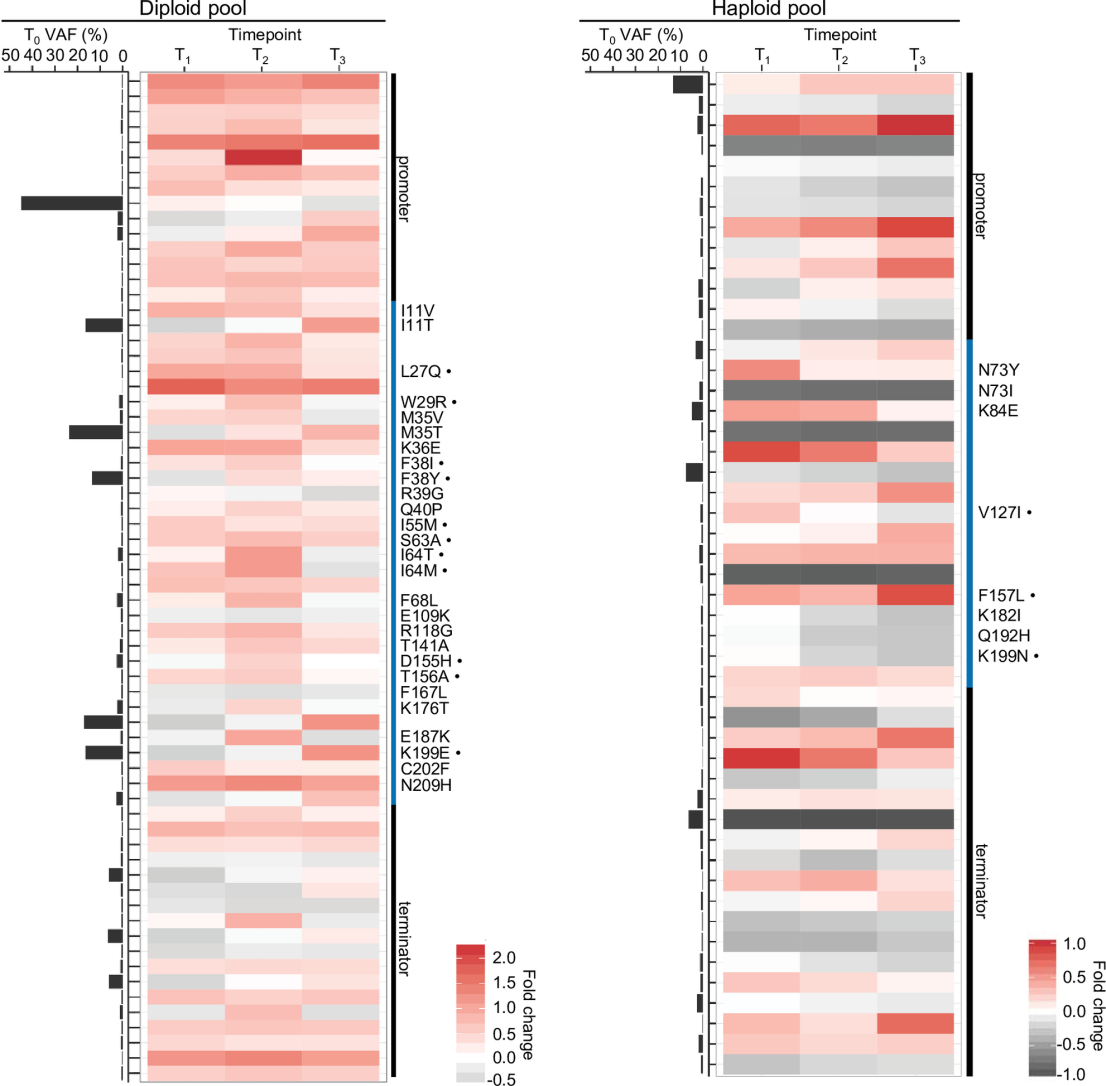
Single nucleotide variant allele frequency estimates for DFR1 loci associated with methotrexate resistance. Estimated variant allele frequencies (VAFs) are shown for DFR1 loci that have a significantly different VAF compared to the starting T0 time point (as described in Methods). Each VAF estimate is based on a sample size of n = 3 following the sample collection design in S2 Fig. Loci are depicted along the rows and time points along the columns. The heatmap shows the relative change in the VAF at each time point compared to T0, in the diploid (left panel) and haploid (right panel) pools. The estimated VAF at T0 is shown along the left side of the heatmap. The promoter and terminator are delineated from the coding sequence by black and blue bars respectively to the right of the heatmap. Non-synonymous mutations are indicated by the wild-type and variant residue. A bullet beside the residue indicates that the locus is conserved with hDHFR.

We used RVD2 to compare the non-reference read rate at the starting time point “T0” to that of all later time points (T1, T2, and T3). We denote the model’s estimate of the true non-reference read rate at each locus the Variant Allele Frequency (VAF) at that locus. This analysis identified 66 variant positions in the *DFR1* locus in the diploid pool and 49 variant positions in the haploid pool (Fig 2; S2 and S3 Tables; and S3 Fig). Among the 35 (53%) coding mutations in the diploid pool, 28 were missense mutations. Exactly 11 of these 28 mutations (39%) correspond to highly conserved residues (Fig 2; S2 Table; and S3 and S4 Figs). We noted that missense mutations that affect M35V and M35T residues, which were previously shown to affect MTX binding affinity and/or MTX resistance, were recovered in our screen [14] (S2 Table). In the haploid pool in contrast, only 8 out of 17 coding mutations (47%) were found to be missense mutations, 3 of which correspond to residues that are conserved in hDHFR (Fig 2; S3 Table; S3 and S4 Figs).

We estimated the diversity in the diploid and haploid pools at each time point by comparing the number and frequency of the variants under selection to the number and frequency of variants in the background strain, which carries only the wild-type allele on the parental plasmid. This procedure accounts for changes in the number and relative abundance of variant alleles, and sequencing variation (see Methods for details). Because the wild-type allele-bearing strain was only sequenced with one replicate, the sensitivity of RVD is low and few variants were called. We record a—when fewer than two variants were called at a time point and no diversity score can be computed. For the haploid strain, the diversity scores for T0 - T3 were 1.37, -, -, - respectively. For the diploid strain, the diversity scores for T0 –T3 were -, 9.20, 7.13, 4.66 respectively. The diversity of the haploid strain is lower than the diversity of the diploid strain at any time point, as would be expected due to the viability requirement imposed on any haploid allele. The diploid diversity score decreases monotonically from T1 to T3. These results align with our expectation that diversity is higher for the diploid pool than the haploid pool and that diversity decreases with time under drug selection.

The nucleotide coding sequence positions called in the diploid and haploid backgrounds do not overlap, except for one (627T>C) (S2 and S3 Tables). We reasoned this is likely due to the random genetic drifts introduced by the sporulation and haploid conversion events (see Methods for details). Furthermore, two positions in the coding sequence identified in the diploid strain showed two minor allele changes over the MTX timecourse e.g. 187T>G (at T1 time point) and 187T>A (at T2 time point), which result in non-synonymous mutations S63A and S63T, respectively (S3 Fig and S2 Table). The emergence of all of these nucleotide changes at given time points suggests that these silent and non-synonymous mutations can have marginal effects in modulating MTX resistance in the diploid genetic background.

We compared the spectrum of variants in the haploid pool to those in the diploid pool prior to selection (T0), because *dfr1* mutants that survive conversion to the haploid state must be viable. We found some *DFR1* positions had high variant allele frequencies (VAFs) in the diploid and haploid pools (Fig 2). The pre-existing *dfr1* haploid allele frequency did not predict the emergence of MTX-resistant variants in later time points (Fig 2; S3 Fig; and S3 Table). In contrast, in the diploid state, 5 out of 6 positions with high initial VAFs (over 10%) increased in abundance upon MTX exposure (Fig 2; S3 Fig; and S2 Table). These observations are consistent with a model in which pre-existing mutations required for viability are no more likely to confer drug resistance, while resistant alleles can be found as pre-existing in the diploid state. Our strategy of surveying *dfr1* alleles in both diploid and haploid backgrounds allowed us to distinguish *dfr1* mutations with dominant resistance phenotypes regardless of whether or not such pre-existing mutants with competitive fitness advantages were already present.

### Validation of methotrexate-resistant *dfr1* alleles

**Fig 3:**
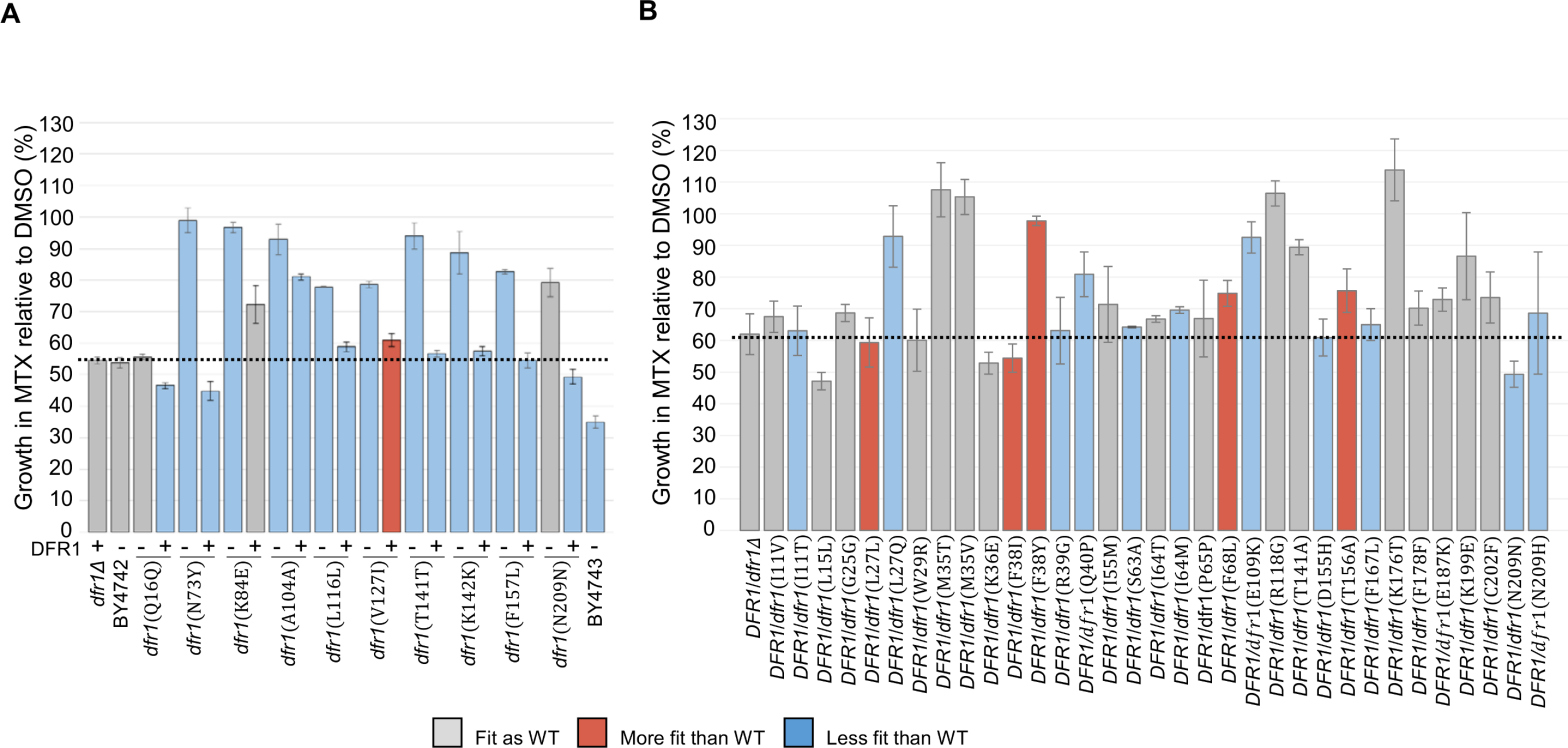
Validation of methotrexate resistant dfr1 mutations. The average fitness of the candidate *dfr1* haploid (A) and diploid (B) mutants upon exposure to MTX (1 mM sublethal dose) or DMSO solvent (2% v/v) were evaluated over 24 hours in a Tecan shaker-reader at 30°C. Mutant alleles expressed in the presence of a wild-type *DFR1* copy are listed with (+). Mutants are colored according to their relative doubling time in comparison to the wild-type strains: BY4742 (WT, A) and *DFR1*/*dfr1*Δ (WT, B) with: blue for longer doubling time (Slower than WT), grey for comparable doubling time (Fit as WT), and red for shorter doubling time (Faster than WT). The average growth under MTX conditions of the control strains are indicated with a dashed line. Error bars indicate standard error, n = 3.

To validate the alleles identified by our high-throughput parallel assay as individual variants, all mutant alleles were reconstructed *de novo* and assayed for MTX resistance in individual growth assays (Methods). We selected all of the coding (non-synonymous and silent) mutations that increased in VAF at the earliest time point (S2 and S3 Tables) and integrated a full length synthetic gene into the chromosomal *DFR1* locus of the isogenic *DFR1*/*dfr1*Δ or *dfr1*Δ strains, such that these alleles were under the control of the endogenous promoter (Methods). All of the *DFR1* mutations in the haploid background were viable, however 6 out of 10 *dfr1* mutants exhibited a slow growth phenotype (Fig 3A and S5 Table). Also, 9 out of 10 *dfr1* alleles (not Q16Q) were reproducibly resistant to a sublethal dose of MTX (Fig 3A; Methods). The majority of the alleles (7 out of 10) are recessive based on our observation that they were no longer MTX resistant when a wild-type *DFR1* copy was expressed (Fig 3A; Methods). In the diploid background, 10 out of 27 *DFR1*/*dfr1* mutants that express non-synonymous mutations were confirmed to exhibit strong resistance to MTX, with at least 80% retained growth relative to each corresponding DMSO-treated strain (Fig 3B). Of these 10 *DFR1*/*dfr1* mutants, 2 exhibit competitive fitness advantages while 3 represent hypomorphic alleles, (with predicted *DFR1* catalytic defects) given their inability to survive under obligate respiratory conditions [40] (Fig 3B; S4 Table and Methods). These results, combined with the experimental differences between initial screen vs. validation experiments (e.g. re-screened individually vs. growth in competitive mixed culture) (Methods) suggest that some of the *DFR1* mutations with marginal MTX resistance, including I55M, F68L, T156A, F178F, E187K and N209H might manifest resistance only when expressed in combination.

### Structure-function relationship of the resistance *dfr1* mutations

**Fig 4:**
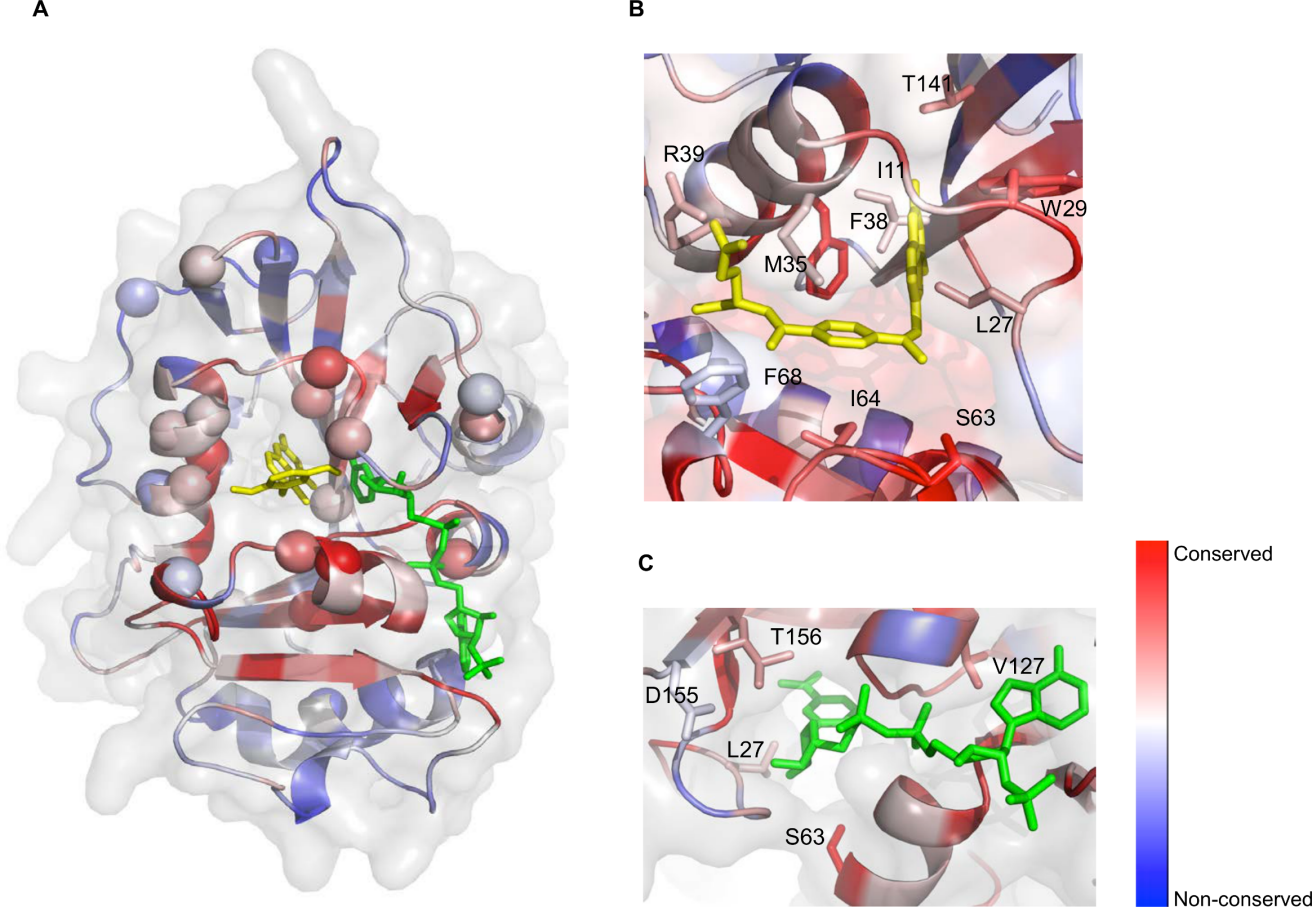
Mapping of the *dfr1* mutations onto the yeast DFR1 model. (A) Cartoon representation of the structural model for the yeast DFR1, colored by sequence conservation (red is conserved, blue is divergent), with surface shown in transparency. The identified resistance point mutations are indicated with a sphere. MTX is highlighted in yellow, and NADPH in green. Mutations largely cluster around conserved residues near the active site of the enzyme. The *dfr1* mutations of residues interacting with MTX (yellow, B) or NADPH (green, C) are mapped onto the yeast DFR1 model. The represented protein is colored by sequence conservation as in (A).

**Fig 5:**
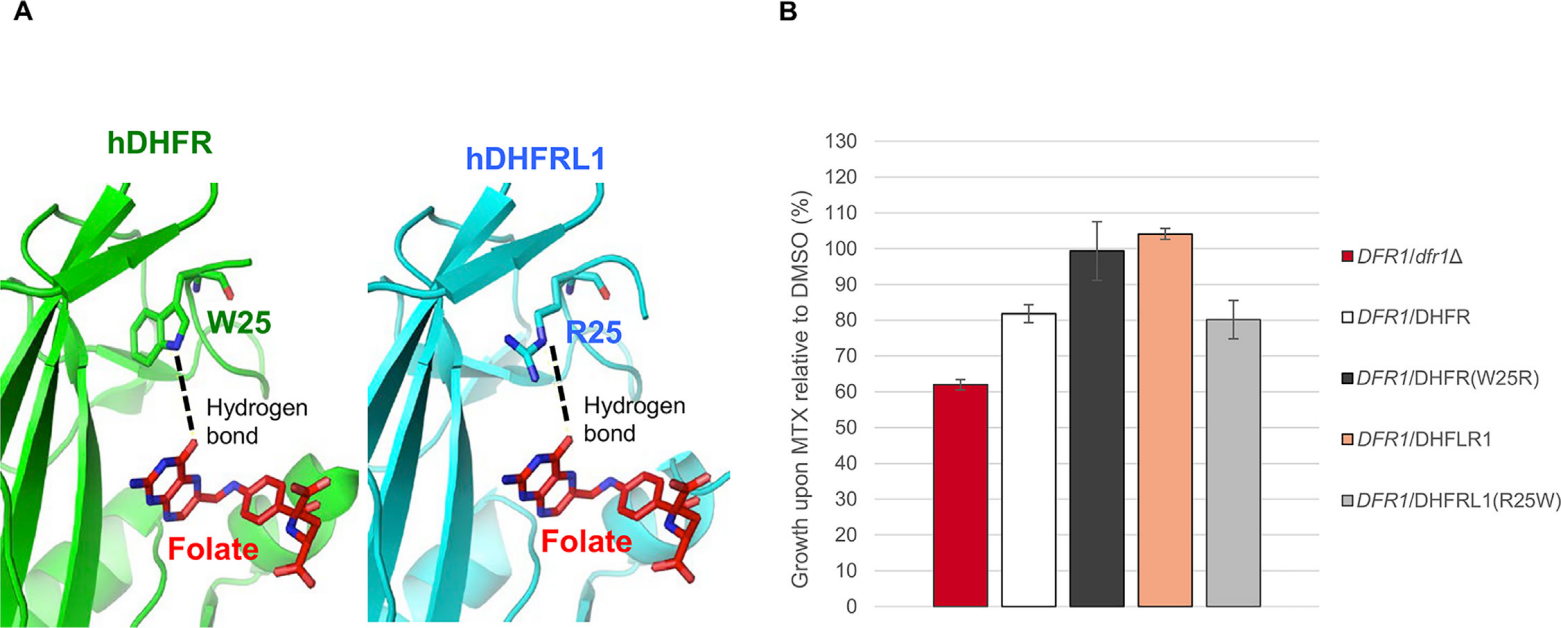
Expression of hDHFR and hDHFRL1 in *DFR1*/*dfr1* heterozygote yeast. (A) The hDHFR and hDHFRL1 active sites in complex with folate (in red; PDB ID 4M6K). The conserved W25 residue in hDHFR forms a hydrophobic interaction stabilized by a hydrogen bond with folate (left panel). In contrast, R25 in hDHFRL1 cannot form hydrophobic interactions with folate, but the stabilizing hydrogen bond is still present (right panel). The side chains of W25 and R25 residues are shown in sticks. (B) The fitness of yeast mutants upon exposure to MTX (1 mM sublethal dose) or DMSO solvent (2% v/v) were evaluated over 24 hours in a Tecan shaker-reader at 30°C. Error bars indicate standard error, n = 3.

Ten of the validated MTX-resistant *dfr1* alleles cluster in the functional binding pocket for folate, the substrate of Dfr1p, or its NADPH cofactor (Fig 4). Specifically, mutations in L27, M35, F38 and T141 correspond to residues that directly interact with MTX (or folate) in hDHFR (Fig 4B and 4C). We hypothesize that these mutations likely reduce MTX affinity to render the drug ineffective. Similarly, mutations found in V127 and T156 correspond to residues situated in the NADPH binding cleft (Fig 4C). Previous work has shown that specific *C*. *galbrata* DHFR inhibitors act by displacing the NADPH cofactor [41,42], suggesting that a similar mechanism could be at work for the V127 and T156 mutations identified in our screen. Other non-synonymous mutations identified in our screen, including F38Y, M35T and M35V, have also been previously reported to lead to MTX resistance [30,32,33,35]. Further, we identified a W29R mutant, a residue known to be essential for enzyme function [43]. Specifically, the side chain of W25 (W29 in yeast) forms hydrophobic aromatic stacking interactions with both MTX (PDB ID 1U72)[10] and folate (Fig 5A) (PDB ID 1DHF)[44]. In the latter case, the protein-ligand interaction is further stabilized by a hydrogen bond formed between the N^ɛ^ of W25 and the hydroxyl group of the folate pteridine ring. We also noted that a Trp residue is conserved at this position, with the notable exception of the recently-identified hDHFR-like 1 (hDHFRL1) protein [45], which has an Arg residue at this position (S3 and S4 Figs). This is the same substitution that we obtained in our screen (W29R) (Fig 2 and S2 Table). An arginine at this position is unable to form the key hydrophobic interactions with MTX [46] and therefore we hypothesized that hDHFRL1 may be resistant to MTX. To test this and to explore the possibility that resistance arises from destabilizing hydrophobic contacts with antifolate [46] (Fig 5A), we investigated the growth fitness of a wild-type human hDHFRL1 construct bearing an arginine at position 25 in a *DFR1*/*dfr1*Δ heterozygote strain (Methods). This change did indeed render the cells resistant to MTX (Fig 5B and S6 Fig). To extend this observation, we constructed a hDHFRL1 construct containing the putative loss-of-resistance allele, R25W, in a *DFR1*/*dfr1*Δ heterozygote strain (Fig 5B and S6 Fig). Although this mutant *DFR1/*hDHFRL1 (R25W) has a comparable growth rate to its wild-type counterpart *DFR1/*hDHFRL1, MTX resistance was abolished by introducing the R25W allele. Conversely, we tested the growth fitness of a hDHFR construct bearing the same mutation W25R (equivalent to W29R in yeast) in a *DFR1*/*dfr1*Δ heterozygote strain (Methods). This human W25R variant was reproducibly MTX resistant (Fig 5B). Of note, the W29R mutant in a yeast construct did not yield MTX resistance in our validation assay (Fig 3B), likely due to the weak ability of W29R variant to persist and modulate MTX resistance in the pool over the timecourse (Fig 2 and S3 Fig). Future work will address the mechanistic differences between the various MTX-resistant *dfr1* alleles and their implications for folate metabolism.

## Discussion

Here we report a combined experimental and statistical approach capable of rapidly achieving high coverage of a targeted region to reliably identify bona fide drug resistant variants of dihydrofolate reductase. As a unique result of this strategy, we achieved increased throughput, resolution and sensitivity critical to detect *dfr1* point mutation alleles that emerge or persist upon exposure to lethal MTX conditions over time. Although we cannot demonstrate we have exhausted all possible resistance conferring *dfr1* mutations in the original *dfr1* variomics library, our innovative approach proves to advance our understanding of the molecular basis of MTX resistance with the identification of a significant fraction of the resistance *dfr1* variant space previously unknown in *S*. *cerevisiae*. By performing parallel competitive screening on diploid and haploid *dfr1* libraries, we also uncovered pre-existing *dfr1* hypomorphic alleles in the diploid state, which are as likely to modulate MTX resistance as haploid *dfr1* mutations with dominant phenotypes. These observations further validate the relevance of interrogating gene variants in the diploid background given that in a human therapeutic target, mutations in only one allele suffice to provide drug resistance.

Our variant calling algorithm, RVD2, is specifically designed to call rare variants in pooled sequencing data. It does so by leveraging replicates of a sample to estimate a baseline error rate at each locus. Then the test sample error rates are compared, in a hypothesis testing framework, to the locus-specific baseline error rate. In previous work, we compared the performance of RVD2 to state-of-the-art variant calling methods using *in vitro* mixtures of synthesized DNA fragments at defined fractions. These experiments showed that RVD2 has higher sensitivity and specificity for detecting rare variants and equivalent sensitivity and specificity for higher frequency (more than 10%) variants.

We also identified the emergence of rare variant alleles, at starting frequencies lower than 1% that are capable of conferring resistance to MTX over time. As variant diversity is lost upon lethal MTX selective pressure, many of these rare alleles do not persist in the pooled conditions suggesting that the presence of epistatic mutations that can affect the evolution trajectory of adaptive MTX resistant alleles [47].

In addition, several novel functional MTX-resistant *dfr1* alleles that disrupt the conserved active-site residues were identified *in vivo*, providing additional genetic insights into the determinants of MTX resistance. Importantly, we show that the yeast-based assay used here is capable of interrogating functional homologs such as the human enzyme. Out of 42 Dfr1 residue changes identified in our screen, 14 (33%) conserved residues are known to display key interactions with antifolate compounds and/or NADPH cofactor, which can affect the potency and selectivity of antifolates [29,33,35,42,48,49]. The remaining 28 (67%) residues identified are novel sites capable of modulating MTX resistance. The catalytic function of such DHFR mutations remains to be explored.

With current ever-improving gene synthesis approaches, determining the consequences of non-coding SNPs will become tenable as will assessing the concomitant effects of causative *dfr1* mutations when expressed in combination. As sequencing technology becomes cheaper and more practical, this platform should in principle be extensible to uncovering linked mutations in small drug targets like DHFR that can confer specific resistance in yeast to address the growing problem of resistance to otherwise effective compounds and FDA drugs.

## Methods

### Yeast strains and drugs

The homozygous diploid reference strain BY4743 *MAT*a (*his3*Δ*1 leu2*Δ*0 LYS2 met15*Δ*0 ura3*Δ*0*)/*MAT*alpha (*his3*Δ*1 leu2*Δ*0 lys2*Δ*0 MET15 ura3*Δ*0*) was used to determine the MEC_100_ (minimum effective concentration to cause 100% cell killing) of methotrexate (MTX) to use for resistance screening and validation growth assays. For MTX resistance screens, a *dfr1* variomics library was used [50]. MTX was purchased from Sigma (M9929) and single-use MTX aliquots were prepared by dissolving MTX in DMSO solvent to 100 mM and stored at -80°C. To counterselect cells carrying the plasmid-borne *dfr1* mutations, 5-Fluoroorotic acid (5’-FOA; Sigma F6625) was added to the media at a final concentration of 1 g/L. To select *DFR1*/*dfr1* cells with the correct *dfr1* integration event, growth sensitivity to G418 sulphate (Geneticin) was verified by adding G418 (Fisher 142480) to a final concentration of 400 mg/L to YPD agar plates.

### Growth assays

For assessing growth fitness in the presence of MTX, yeast strains and pools were cultured to mid-log phase (OD_600_ ~0.5) in synthetic complete (SC) liquid media before adjusting the cultures to an initial OD_600_ of 0.0625. Cells were then transferred to a 96-well microtiter plate containing liquid SC media with either MTX or DMSO solvent (2% v/v) as control. To determine the MEC_100_ dose of MTX, a range of doses (0.025, 0.05, 0.1, 0.3 and 2 mM) were tested against the wild-type BY4743 strain. Cell growth upon MTX treatment relative to DMSO solvent was assayed in three biological replicates using a spectrophotometer Tecan shaker-reader that measured OD_595_ values over 24 hours at 30°C. Cell growth was inhibited at ~100% at 2 mM, which was the determined lethal dose for the MTX resistance assay (S1 Fig). To confirm MTX resistance, the reconstructed yeast strains were cultured in rich YPD media at a sublethal dose (1 mM) of MTX. Cell growth upon MTX treatment relative to DMSO solvent was assayed in three biological replicates using a Tecan shaker-reader over 24 hours at 30°C. For each mutant, the percent of growth rate in MTX relative to DMSO was calculated and the average and standard error of three biological replicates reported in S4 and S5 Tables. Mutant strains that showed a reproducible growth in the presence of the drug were confirmed to be true MTX-resistant strains (Fig 3). Cell growth in obligate respiratory media was used as a proxy to assess mitochondrial folate metabolism. *Dfr1* mutant cells with non-synonymous mutations were cultured in obligate respiratory growth media using YPG media prepared with a non-fermentable carbon source (glycerol at 3% v/v). Growth or lack of growth in YPG media was assayed in three independent assays. Cultures that displayed two doublings or fewer after 24 hours in YPG were scored as respiratory-defective. The respiratory-proficient strains *DFR1*/*dfr1*Δ and wild-type BY4742 were included, in parallel, as controls.

### Manipulation of the *dfr1* functional variomics library

The starting *dfr1* variomics library consists of at least 2 x 10^5^ independent *dfr1* variant alleles, with single and multiple point mutations per allele. The variomics library is cloned into a CEN-based plasmid under control of native upstream and downstream regulatory regions, and transformed into a *DFR1*/*dfr1*Δ heterozygote convertible diploid strain [50]. The diploid variomics pool is cultured in synthetic dropout medium lacking uracil (SD-URA) at 30°C to generate a working stock of OD_600_ 1, equivalent to an average of 50-fold coverage (independent cells) for each of the 2 x 10^5^ independent variants. The latter calculation assumes that each *DFR1*/*dfr1*Δ cell harbors one variant *dfr1* allele and that all doubling times are similar. To generate a *dfr1* pool in haploid *MATa* cells lacking the chromosomal wild-type *DFR1* gene, the *dfr1* diploid pool was sporulated and subsequently haploid converted using the previously described optimized procedures [16], with the following modifications. Diploid cells were cultured in 200 ml sporulation medium at room temperature with vigorous shaking (200 rpm) for 5 days in the dark to increase sporulation. ~ 2 x 10^5^ sporulated cells, with an average 10-fold variant coverage were cultured in 400 ml haploid selection medium to enrich for *MATa dfr1* G418R URA^+^ haploid cells at 30°C for 2 days [51]. Sporulation and haploid conversion recovered a *dfr1* haploid pool with ~11% of the initial *dfr1* alleles, which represents a total of 2.2 x 10^4^ viable *dfr1* alleles. The selection for haploid cells and genetic “bottle necks” introduced in the methodology (Fig 1 and S2 Fig) are likely to exert additional selective pressure on the haploid pool. Hence, we predict a smaller proportion of MTX-resistant variants that sustain cell viability is present in the haploid pool.

### Methotrexate resistance screening assay

The *DFR1*/*dfr1*Δ diploid and *dfr1*Δ haploid pools were cultured to an initial OD_600_ of 0.01, with an average 10-fold variant coverage observed in the *dfr1* variomics library. Each pool was screened in triplicate wells (n1, n2, n3 technical replicates) in 6-well microtiter plates over a 6-day time course in 10 ml of SD-URA media supplemented with MTX at 2 mM (MEC_100_) (Fig 1 and S2 Fig). Media supplemented with DMSO (2% v/v) was prepared in parallel as a negative control. The screening assay consists of 3 time points, where the MTX and DMSO treated pools were propagated at 30°C with vigorous shaking (200 rpm). Sampling was done every 2 days, which typically resulted in 8-generation propagation for the DMSO-treated pools (S2 Fig). The first replicate (n1) set of cultures for MTX and DMSO was used for propagating the subsequent time point: at each time point, MTX-treated cells from the n1 replicate well were diluted to OD_600_ 0.01 with fresh SD-URA medium supplemented with either MTX or DMSO, and transferred to the equivalent 3 replicate wells in a new microtiter plate (S2 Fig). At each time point, MTX-treated cultures from all replicate wells were harvested for *DFR1-*targeted sequencing and analysis (see below). The initial *dfr1* variomics libraries of both diploid and haploid pools (time point 0) were also split in three technical replicates to assess sample-to-sample variation. The sample size per condition (n = 3) and expected sequencing median read depth (20,000x) was selected based our published power analysis from a previous version of our statistical model [52] and experience with this version of the model [14] to detect a minimum variant allele frequency of 0.1%.

### Plasmid extraction and PCR reactions

Plasmids were extracted from harvested MTX-treated cell pools at each time point using the DNA extraction protocol described previously [50]. PCR reactions were performed using Phusion High Fidelity polymerase, according to the manufacturer’s instructions (Thermo Fisher Scientific) with the following modifications. To amplify the *dfr1* amplicons from the plasmid-containing extracts, PCR reactions were performed in 50 μl containing 100 ng of plasmid extract and universal plasmid-specific oligonucleotides at 0.5 μM [16]. The cycling protocol was as follows: 1× (98°C for 30 sec), 30× (98°C for 10 sec, 52°C for 30 sec, 72°C for 45 sec),1× (72°C for 5 min). For colony PCRs, a fraction of each yeast colony was picked using a plastic micropipet tip and placed at the bottom of the reaction tube containing 10 μl of 20 mM NaOH. Samples were boiled for 5 min and 1 μl of each sample was used for the PCR reactions in a total of 25 μl containing oligonucleotides (2.5 μM). For a complete list of oligonucleotides used, see S1 Table. The cycling protocol for colony PCR amplification was as follows: 1× (98°C for 30 sec), 30× (98°C for 10 sec, 48°C for 30 sec, 72°C for 10 sec),1× (72°C for 5 min). All reaction products were analyzed on a 1% (w/v) agarose gel.

### Deep sequencing of MTX-treated *dfr1* pools by seqWell

*Dfr1* amplicons prepared by PCR were first purified using the Thermo Fisher Scientific PCR purification kit, according to the manufacturer’s instructions, quantified using Qubit fluorometry (Life Technologies) and diluted for sequencing library preparation. Libraries were constructed using plexWell library kit technology (seqWell, Beverly MA). In this approach, each 1+ kb pool of diverse amplicons is tagged with a pool-specific barcode via a transposase-mediated adapter addition at random locations. After this tagging, the pools of amplicons are then pooled into a single meta-pool, and subjected to a second transposase-mediated adapter addition. Fragments of this pool containing sequence from each of the two iterative adapter additions are then amplified to yield a final sequence library representing identifiable fragments from each original amplicon pool. Sequencing data is available upon request. We have deposited the raw fastq files at the NCBI SRA under the accession number SRP072709.

### Variant allele frequency estimation

*DFR1*-targeted sequencing data was collected and processed separately for the diploid and haploid experiments. To align the raw (fastq) sequencing data to the *S*. *cerevisiae* genome, we first trimmed the Illumina adaptor sequences using cutadapt (v 1.7.1) (--anywhere AGATCGGAAGAGC). Then the paired-end reads were aligned to the April 2011 UCSC *S*. *cerevisae* reference genome (sacCer3) using bwa (v 0.7.12) mem with the -M flag set. Finally, the resulting bam files were indexed and sorted for subsequent processing and visualization.

First, we used samtools (v 1.2) mpileup to generate pileup files for the *DFR1* gene region chrXV:780367–782084. We also set the -A and -BQ0 flag to get high quality read depth estimates without discarding anomalous reads, and we set the maximum depth to 10,000,000 to ensure no truncation of read depth occurs. We used a custom program, described previously [14], to provide the count of each base pair at each position in the region of interest. Then, we ran RVD2 gibbs on the wild-type, T0, T1, T2, and T3 data sets separately with the default warm-up, thinning and sample size parameters to estimate the model parameters and latent variables in the RVD2 statistical model. Finally, we called variants between all pairs of data sets using RVD2 somatic test with an interval of [0.001, 100] and a significance level (α) of 0.05. The somatic test calls a provisional variant at a position if the Bayesian posterior probability (estimated from a sample size of 1000 from the model posterior distribution) that the difference between the VAF in two data sets (e.g. T0 and T1) is in the interval is greater than 1-α (two-sided). The provisional variant is called a variant if the distribution over the non-reference bases is non-uniform by a chi-squared test with a significance level of 0.05. Calls based on these posterior credible intervals were not adjusted for multiple comparisons and we did not detect any gross deviations from the assumptions of the statistical model for this data. Further details on the estimation procedures and hypothesis test are provided in the RVD2 study [14].

### Code availability

Our statistical model and variant calling method as described previously [14] is publicly and freely available at https://bitbucket.org/flahertylab/rvd.

### Diversity estimation

Given a set of called variants, *V*, we compute the diversity as follows. To compute the diversity, we first compute the KL divergence from ${\hat{\mu }}_{j}$ to *p* where *p* = 1/|*V*| where ${\hat{\mu }}_{j}$ is the estimated non-reference read rate at called position *j*. The KL divergence is zero if and only if ${\hat{\mu }}_{j}$ is equal to *p*. In that case, each variant is equally represented in the pool and the entire pool was made up of variant clones. Otherwise, the KL divergence is greater than zero. We compute the diversity as

$$D=\sum_{j\in V}1+\tanh D_{KL}(p\|{\hat{\mu }}_{j}).$$

The tanh function is -1 when $D_{KL}\left(p\|{\hat{\mu }}_{j}\right)=\infty$ and 0 when $D_{KL}\left(p\|{\hat{\mu }}_{j}\right)=0$. So, $1+\tanh D_{KL}\left(p\|{\hat{\mu }}_{j}\right)$ is 0 when $D_{KL}\left(p\|{\hat{\mu }}_{j}\right)=\infty$ and 1 when $D_{KL}\left(p\|{\hat{\mu }}_{j}\right)=0$. Summing over all of the called variants means that the maximum value of the diversity grows with the number of variants. Therefore, this diversity measure captures both the uniformity of the distribution of the variants as well as the total number of variants.

### IDT gene fragments for the construction of *dfr1* mutants

Gene fragments containing coding sequence point mutations flanked by *DFR1* specific homology sequences were synthesized by IDT (S6 Table). Gene fragments containing wild-type yeast *DFR1* and human DHFR and DHFRL1 sequences were included as controls. Each gene fragment was resuspended in water (molecular grade, Thermo Fisher Scientific) to make a 10 ng/μl stock and stored at -20°C. Prior to yeast transformation, the gene fragments were PCR amplified using Phusion High Fidelity, according to the manufacturer’s instructions (Thermo Fisher Scientific). For each PCR reaction, 10 ng of the IDT gene fragment was used as template in a 50 μl reaction and amplified with the oligonucleotides listed in S1 Table.

### Construction of *dfr1* mutants by yeast transformation

To confirm MTX resistance, each *dfr1* point mutant fragment was integrated into the *dfr1*:: *kanMX* locus of the haploid and diploid progenitor strains, which harbours the *dfr1* variomics library. The diploid *DFR1/dfr1*Δ progenitor strain was first outgrown in YPD containing 5’-FOA for 2 days at 30°C to counterselect for the Ura^+^ *dfr1* plasmids prior to the transformation. The haploid *dfr1* strain was propagated in SD-URA medium to maintain the plasmid-borne *dfr1* pool in order to maintain its viability. A high efficiency transformation protocol was used to create the mutants by mitotic recombination [53]. The progenitor strains were first cultured to mid-log phase in liquid SD-URA media and subsequently transformed with the *dfr1* fragments according to the standard heat shock protocol [53]. Human DHFR and DHFRL1 point mutations were also integrated into the *dfr1*::*kanMX* locus to generate *dfr1* yeast hybrid strains. To confirm that the yeast transformants have the correct integration, both diploid and haploid clones were 1) confirmed for the appearance of PCR products of the expected size using oligonucleotides that span the upstream and downstream junctions of the *dfr1*:: *kanMX* locus (S1 Table); 2) confirmed for loss of G418 resistance; and 3) confirmed for MTX resistance using a sublethal dose of MTX in liquid growth assays (Fig 3). Additionally, the haploid clones were counter-selected in 5’-FOA containing YPD agar plates to kill any cells carrying the plasmid-borne *dfr1* mutations and confirmed for the absence of plasmid-borne *dfr1* PCR products by colony PCR using plasmid specific oligonucleotides (S1 Table). To make the yeast/human mutant constructs in the heterozygous diploid *DFR1/dfr1* background, the haploid *dfr1* mutants and wild-type BY4741 control were mated with the wild-type haploid BY4742 (*MAT*alpha *his3*Δ*1 leu2*Δ*0 lys2*Δ*0 MET15 ura3*Δ*0*) using standardized yeast manipulation procedures [54]. The diploid constructs were confirmed by selectively growing in agar plates containing synthetic dropout medium that lacks lysine and methionine amino acids (SD-LYS-MET) for 2 days at 30°C.

### Structure modelling and mapping of coding mutations

The multiple sequence alignment for the dihydrofolate reductase protein was obtained with ClustalW [55] and the S4 Fig generated with ESPript [56]. The consensus sequence for all DHFR homologues (S5 Fig) was built using WebLogo [57]. The *Saccharomyces cerevisiae* DFR1 structural model was generated with Modeller [58] using the closest homologue of known structure (*Candida glabrata* DHFR, 54% identity, PDB ID: 3CSE) [41] as a template. The coordinates of NADPH were obtained by superimposing the structure of the *C*. *glabrata* DHFR structure in complex with NADPH (PDB ID: 3CSE), and the coordinates of methotrexate were obtained by superimposing the structure of the *E*. *coli* DHFR in complex with methotrexate (PDB ID: 4P66) [59]. The colour gradient for the sequence conservation was generated with ConSurf [60], using the aforementioned multiple sequence alignment. All structure figures were obtained with PyMol (Schrodinger, LLC).

## Supporting Information

S1 Fig Methotrexate pre-screen against the wild-type strain. The growth fitness of MTX sensitive BY4743 strain was evaluated upon exposure to synthetic complete (SC) media supplemented with a dose range of MTX (0.025–2 mM) and DMSO solvent control (2% v/v) in a Tecan shaker-reader at 30°C. Three independent growth assays were performed. Error bars indicate standard error, n = 3.
(TIF)

S2 Fig Methotrexate resistance screen. To identify MTX-resistant *dfr1* mutants, the starting diploid and haploid pools were cultured at an initial OD_600_ of 0.01 in triplicate wells (n1, n2, n3) of a 6-well microtiter assay plate containing 10 ml of synthetic dropout media lacking uracil (SD-URA) and supplemented with either MTX or DMSO, and grown at 30°C with vigorous shaking. At each timepoint (every 2 d), cultures from all technical replicates were harvested for plasmid extraction as described in Fig 1. After each harvest the n1 replicate was used to propagate the subsequent time point, by diluting cells to OD_600_ 0.01 in fresh SD-URA medium containing either MTX or DMSO, and transferring these to 3 replicate wells in a new microtiter plate.
(TIF)

S3 Fig Dfr1 minor allele frequencies called in the diploid and haploid *dfr1* variomics pools. Variant allele frequency estimates from the RVD2 model are provided as individual.pdf figures for each DFR1 locus. The pool and position (sacCer3 reference) make up the.pdf file title. Each figure shows four time points (T0-T3) along the x-axis and error rate along the y-axis. The filled circles and error bars show that point estimate and posterior 95% Bayesian credible intervals for μj, the locus-specific error rate or variant allele frequency (VAF) in the text, from the model where j indexes the position. The plotted numbers (1, 2, 3) show the locus-specific error rate for the sequencing replicate, θnj in the RVD2 model where n = 1, 2, or 3. These figures illustrate the uncertainty in the measurements due to both finite sequencing depth and reproducibility between replicates captured in the model.
(TIF)

S4 Fig Protein sequence alignment. Multiple alignment of the DHFR protein sequence in species: *Saccharomyces cerevisiae*, *Candida glabrata*, *Homo sapiens* (Homo_DHFR), hDHFR-like 1 (Homo_DHFRL1), *Mycobacterium tuberculosis*, *Escherichia coli*. Conserved residues are in red boxes and similar residues highlighted in red. Secondary structure elements for the *C*. *glabrata* and hDHFR1 (Homo_DHFR) are shown, in green and blue respectively, with the corresponding numbering indicated. The R25 residue that confers MXT resistance in hDHFRL1 is highlighted in purple.
(TIF)

S5 Fig The consensus sequence for DHFR homologues. The conserved residue W25 in hDHFR (W29 in yeast) is indicated with an arrow. The consensus sequence for all DHFR homologues was built using WebLogo.
(TIF)

S6 Fig Growth fitness of *DFR1*/DHFRL1 and *DFR1*/DHFRL1(R25W) strains upon exposure to lethal methotrexate conditions. The fitness of *DFR1*/DHFRL1, *DFR1*/DHFRL1 (R25W) and *DFR1*/*dfr1*Δ strains upon exposure to MTX (2 mM) and DMSO solvent (1% v/v) were evaluated over 30 hours in a Tecan shaker-reader at 30°C. Growth fitness was evaluated in three independent assays and representative profiles are shown.
(TIF)

S1 Table List of oligonucleotides used in this study. (PDF)

S2 Table Variant calls in the diploid *DFR1*/*dfr1*Δ pool. (PDF)

S3 Table Variant calls in the haploid *dfr1*Δ pool. (PDF)

S4 Table The fitness profiles of *dfr1*, hDHFR and hDHFRL1 alleles in the diploid *DFR1*/*dfr1*Δ background. (PDF)

S5 Table The fitness profiles of *dfr1* alleles identified in the haploid *dfr1*Δ background. (PDF)

S6 Table Synthetic gene fragments used to reconstruct *dfr1* mutants. (PDF)
