# Supplementary figures and images for: Reverse Chemical Genetics: Comprehensive Fitness Profiling Reveals the Spectrum of Drug Target Interactions
Source: PLoS Genet. 2016 Sep 2;12(9):e1006275. doi: 10.1371/journal.pgen.1006275 (PMC5010250; doi:10.1371/journal.pgen.1006275)

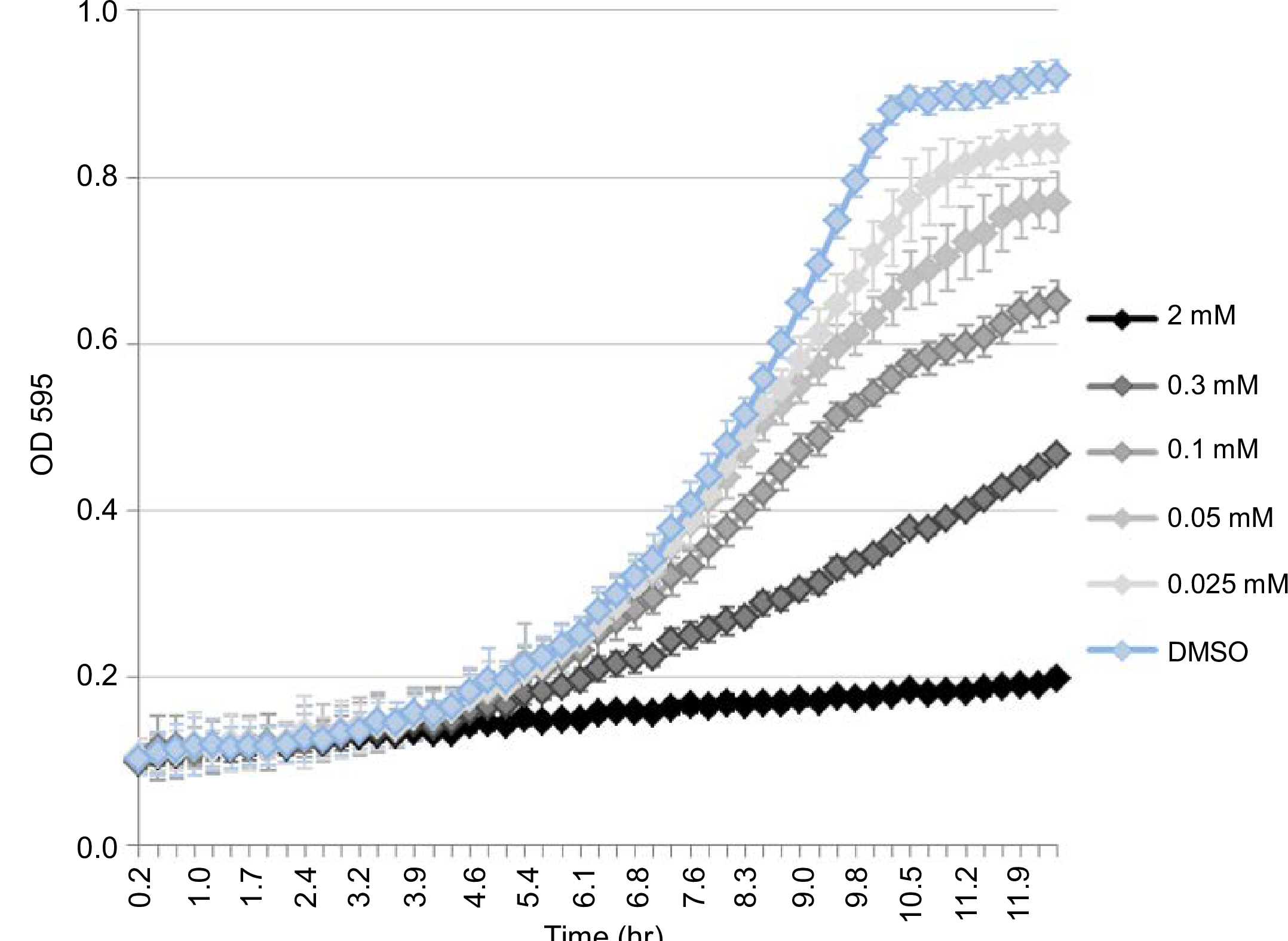

Supplement: S1 Fig — The growth fitness of MTX sensitive BY4743 strain was evaluated upon exposure to synthetic complete (SC) media supplemented with a dose range of MTX (0.025–2 mM) and DMSO solvent control (2% v/v) in a Tecan shaker-reader at 30°C. Three independent growth assays were performed. Error bars indicate standard error, n = 3. (TIF) [file pgen.1006275.s001.tif]

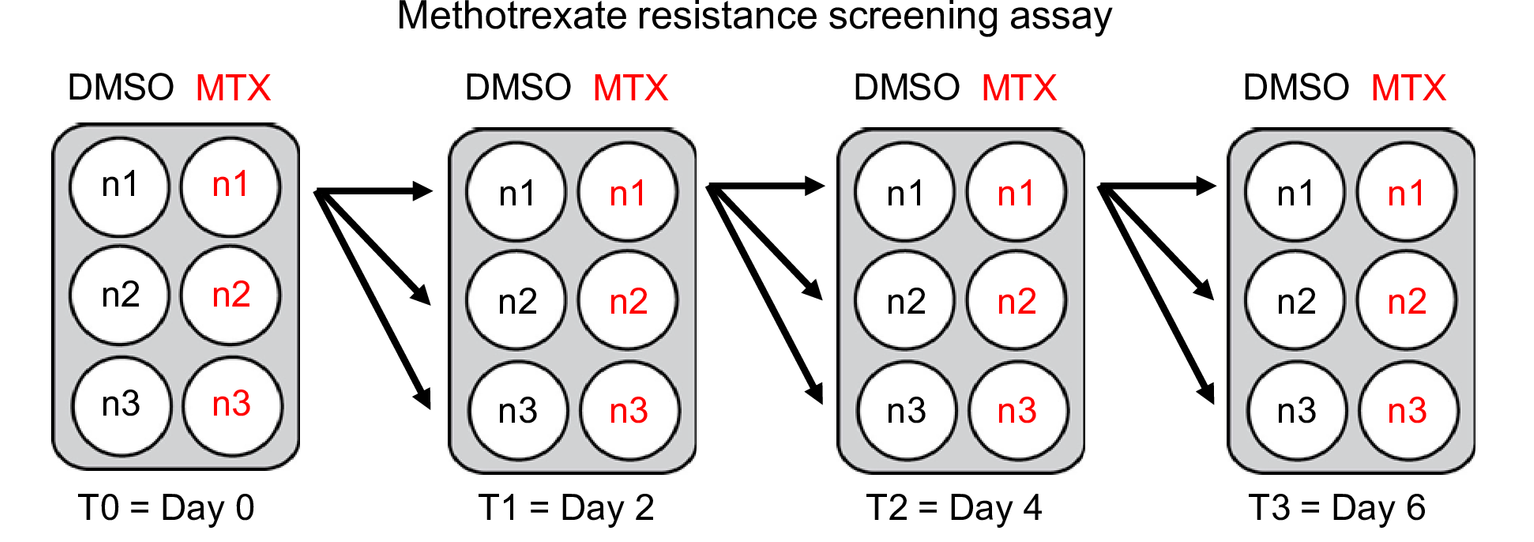

Supplement: S2 Fig — To identify MTX-resistant dfr1 mutants, the starting diploid and haploid pools were cultured at an initial OD600 of 0.01 in triplicate wells (n1, n2, n3) of a 6-well microtiter assay plate containing 10 ml of synthetic dropout media lacking uracil (SD-URA) and supplemented with either MTX or DMSO, and grown at 30°C with vigorous shaking. At each timepoint (every 2 d), cultures from all technical replicates were harvested for plasmid extraction as described in Fig 1. After each harvest the n1 replicate was used to propagate the subsequent time point, by diluting cells to OD600 0.01 in fresh SD-URA medium containing either MTX or DMSO, and transferring these to 3 replicate wells in a new microtiter plate. (TIF) [file pgen.1006275.s002.tif]

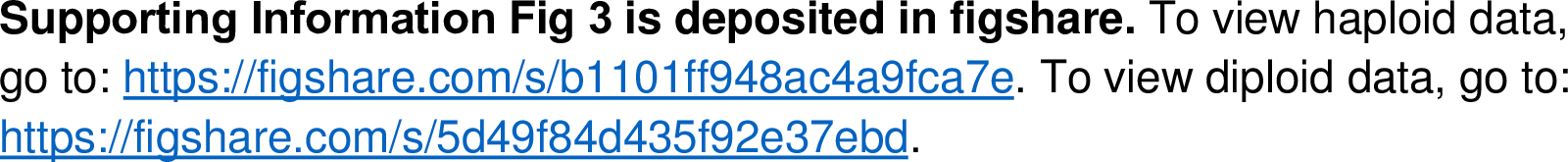

Supplement: S3 Fig — Variant allele frequency estimates from the RVD2 model are provided as individual.pdf figures for each DFR1 locus. The pool and position (sacCer3 reference) make up the.pdf file title. Each figure shows four time points (T0-T3) along the x-axis and error rate along the y-axis. The filled circles and error bars show that point estimate and posterior 95% Bayesian credible intervals for μj, the locus-specific error rate or variant allele frequency (VAF) in the text, from the model where j indexes the position. The plotted numbers (1, 2, 3) show the locus-specific error rate for the sequencing replicate, θnj in the RVD2 model where n = 1, 2, or 3. These figures illustrate the uncertainty in the measurements due to both finite sequencing depth and reproducibility between replicates captured in the model. (TIF) [file pgen.1006275.s003.tif]

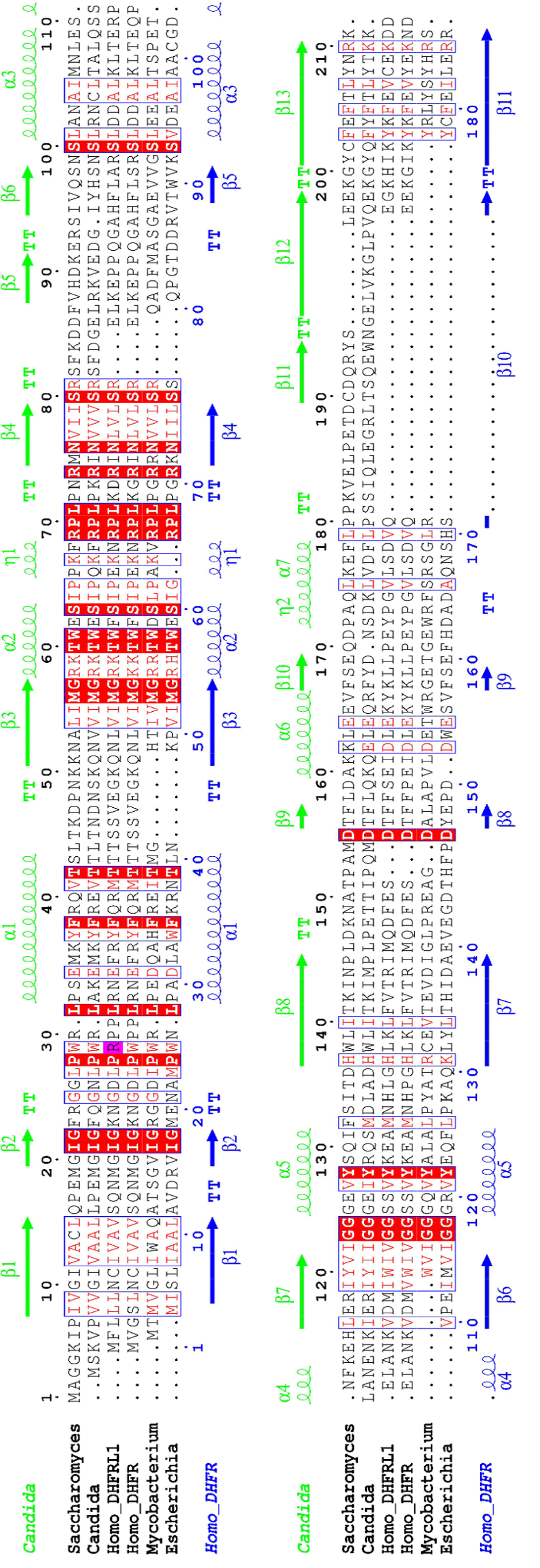

Supplement: S4 Fig — Multiple alignment of the DHFR protein sequence in species: Saccharomyces cerevisiae, Candida glabrata, Homo sapiens (Homo_DHFR), hDHFR-like 1 (Homo_DHFRL1), Mycobacterium tuberculosis, Escherichia coli. Conserved residues are in red boxes and similar residues highlighted in red. Secondary structure elements for the C. glabrata and hDHFR1 (Homo_DHFR) are shown, in green and blue respectively, with the corresponding numbering indicated. The R25 residue that confers MXT resistance in hDHFRL1 is highlighted in purple. (TIF) [file pgen.1006275.s004.tif]

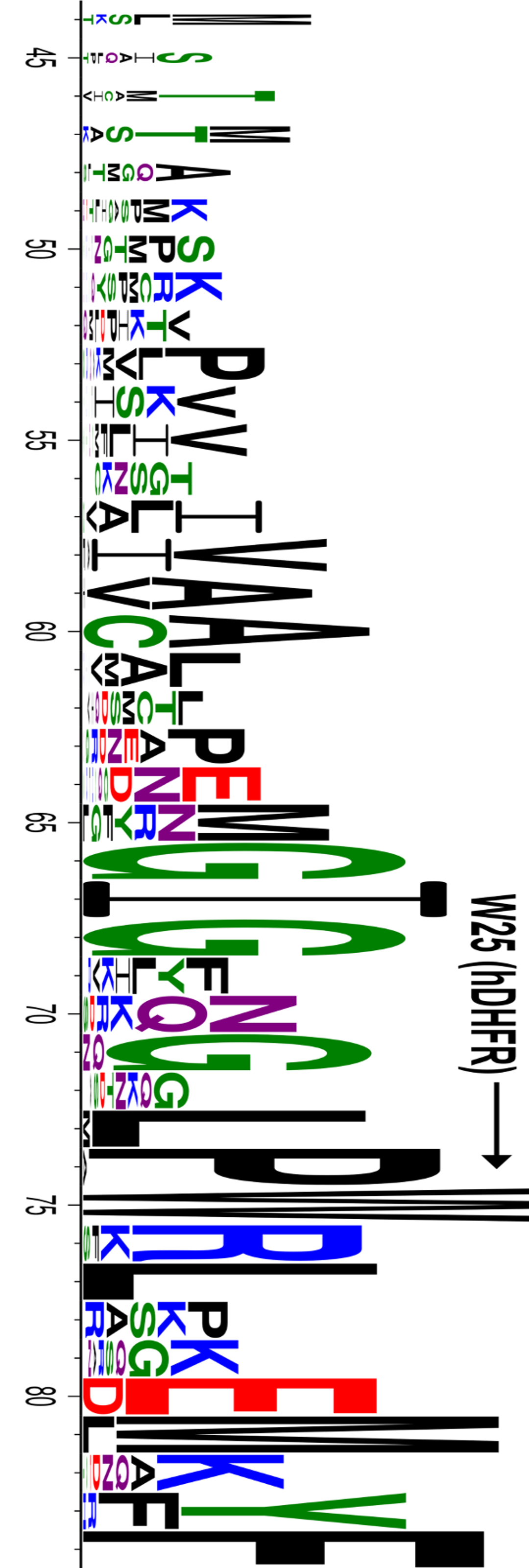

Supplement: S5 Fig — The conserved residue W25 in hDHFR (W29 in yeast) is indicated with an arrow. The consensus sequence for all DHFR homologues was built using WebLogo. (TIF) [file pgen.1006275.s005.tif]

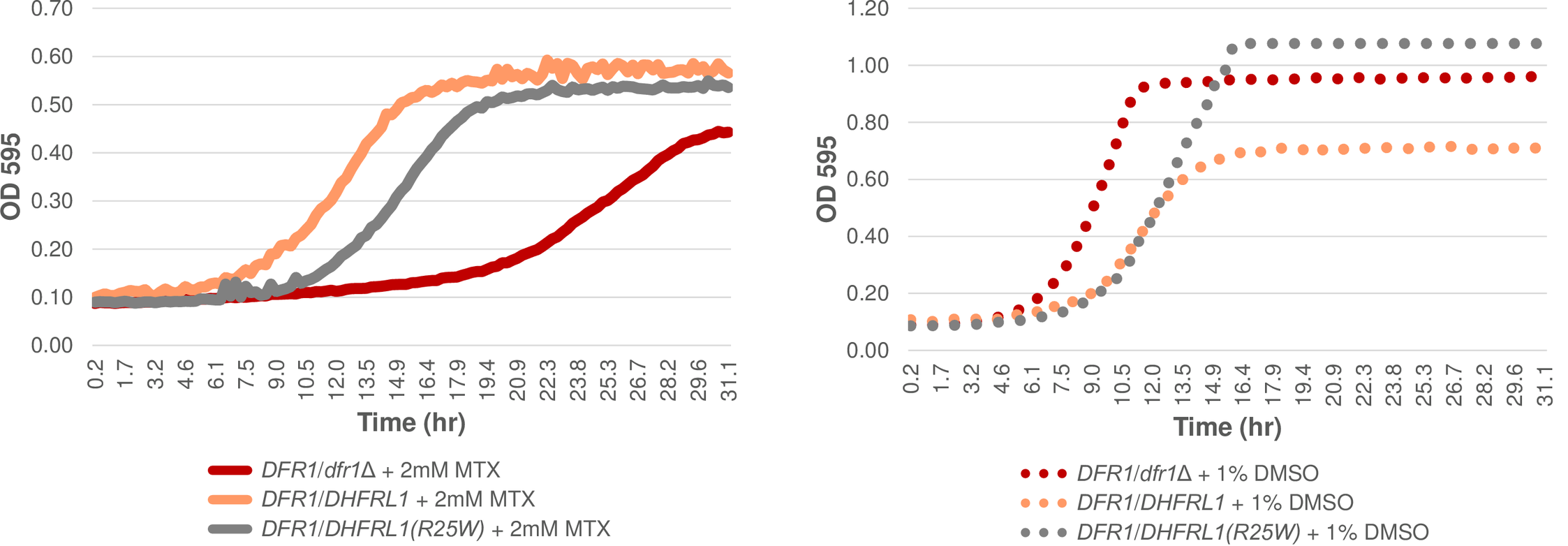

Supplement: S6 Fig — The fitness of DFR1/DHFRL1, DFR1/DHFRL1 (R25W) and DFR1/dfr1Δ strains upon exposure to MTX (2 mM) and DMSO solvent (1% v/v) were evaluated over 30 hours in a Tecan shaker-reader at 30°C. Growth fitness was evaluated in three independent assays and representative profiles are shown. (TIF) [file pgen.1006275.s006.tif]
