# Supplementary material for: Reverse Chemical Genetics: Comprehensive Fitness Profiling Reveals the Spectrum of Drug Target Interactions
Source: PLoS Genet. 2016 Sep 2;12(9):e1006275. doi: 10.1371/journal.pgen.1006275 (PMC5010250; doi:10.1371/journal.pgen.1006275)
Supplement: S1 Table — (PDF) [file pgen.1006275.s007.pdf]

S1 Table. List of oligonucleotides used in this study.

Primer	Sequence (5' to 3')	Notes
1	CCAGGCTTTACACTTTATGCT	Forward primer for plasmid-borne *dfr1* mutant amplification
2	CTGTTGGGAAGGGCGATC	Reverse primer for plasmid-borne *dfr1* mutant amplification
3	ACATTATGCTTTGCATGATA	Forward primer for *DFR1* gene fragment amplification; binds 55-nt upstream of *DFR1* ORF
4	GTTCTATCACTATTTAACATATTAAAAAAAAT ATACGGGCGGAGAGGTTTCATTT	Reverse primer with a 35-nt overhang sequence for *DFR1* gene fragment amplification; binds 14-nt downstream of *DFR1* ORF
5	GTTCTATCACTATTTAACATATTAAAAAAAAT ATACGGGCGGAGAGGTTATCGTT	Reverse primer with a 35-nt overhang sequence for *DHFR* gene fragment amplification; binds 14-nt downstream of *DFR1* ORF
6	GTTCTATCACTATTTAACATATTAAAAAAAAT ATAGTCGTCCTTTTCACAAACTT	Reverse primer with a 35-nt overhang sequence for *DHFRL1* gene fragment amplification; binds 14-nt downstream of *DFR1* ORF
7	TCCTTACGTGCGTGAGAT	Forward primer that binds ~100-nt upstream of the *DFR1* ORF; used for integration boundary validation
8	TGGATTCCCATGTCTTCCTT	Reverse primer that binds within the *DFR1* ORF; used for integration boundary validation
9	AGTATTTAGCGAGCAAGATC	Forward primer that binds within the *DFR1* ORF; used for integration boundary validation
10	AAATACTGCAGGTGAGGC	Reverse primer that binds ~120-nt downstream of the *DFR1* ORF; used for integration boundary validation
