# Supplementary material for: Reverse Chemical Genetics: Comprehensive Fitness Profiling Reveals the Spectrum of Drug Target Interactions
Source: PLoS Genet. 2016 Sep 2;12(9):e1006275. doi: 10.1371/journal.pgen.1006275 (PMC5010250; doi:10.1371/journal.pgen.1006275)
Supplement: S2 Table — (PDF) [file pgen.1006275.s008.pdf]

S2 Table. Variant calls in the diploid *DFR1*/*dfr1*Δ pool.

Position	Region	T1	T2	T3	Residue
780403	promoter	-503C>T	-503C>A	-503C>A	
780414	promoter	-492A>T	-	-	
780415	promoter	-	-491T>G	-	
780431	promoter	-475C>T	-475C>T	-	
780458	promoter	-448C>A	-448C>A	-448C>A	
780467	promoter	-	-439T>C	-	
780531	promoter	-	-375T>G	-	
780618	promoter	-288A>T	-	-	
780619	promoter	-287A>T	-	-287A>T	
780640	promoter	-266T>C	-	-	
780714	promoter	-	-	-192A>G	
780748	promoter	-	-158T>C	-	
780789	promoter	-117C>A	-	-	
780811	promoter	-	-95C>A	-	
780814	promoter	-	-92T>C	-	
780936	cds	31A>G	31A>G	-	I11V [+]
780937	cds	32T>C	-	32T>C	I11T [+]
780950	cds	-	45A>G	-	L15L[+]
780980	cds	-	75A>G	-	G25G[+]
780985	cds	-	80T>A	-	L27Q[+]
780986	cds	81A>G	-	-	L27L[+]
780990	cds	-	85T>C	-	W29R[+]
781008	cds	103A>G	103A>G	103A>G	M35V[+]
781009	cds	104T>C	104T>C	104T>C	M35T[+]
781011	cds	-	106A>G	-	K36E[+]
781017	cds	-	112T>A	-	F38I[+]
781018	cds	113T>A	113T>A	-	F38Y[+]
781020	cds	-	-	115A>G	R39G[+]
781024	cds	-	119A>C	-	Q40P[+]
781070	cds	165A>G	-	-	I55M[+]
781092	cds	187T>G	187T>A	-	S63A[+]/S63T
781096	cds	-	191T>C	-	I64T[+]
781097	cds	192A>G	192A>G	192A>G	I64M[+]
781100	cds	195G>A	195G>A	-	P65P[+]
781107	cds	-	212T>C	-	F68L[+]
781230	cds	-	325G>A	-	E109K[+]
781257	cds	-	352A>G	-	R118G[+]
781326	cds	-	421A>G	-	T141A[+]
781368	cds	-	463G>C	-	D155H[+]
781371	cds	-	466A>G	-	T156A[+]
781406	cds	-	501T>G	501T>C	F167L[+]/F167F
781432	cds	527A>C	527A>C	-	K176T[+]
781439	cds	534T>C	-	534T>C	F178F[+]
781464	cds	-	559G>A	559G>A	E187K[+]
781500	cds	595A>G	-	595A>G	K199E[+]
781510	cds	605G>T	-	-	C202F[+]
781530	cds	625A>C	625A>C	625A>C	N209H[+]
781532	cds	627T>C	-	627T>C	N209N[+]
781588	terminator	-	*47A>G	-	
781597	terminator	*56G>T	-	-	
781616	terminator	-	-	*75G>T	
781683	terminator	-	-	*142C>A	
781697	terminator	*156C>T	-	-	
781706	terminator	-	*165T>C	-	
781720	terminator	-	*179A>C	*179A>C	
781723	terminator	-	*182A>G	-	
781776	terminator	*185T>C	-	-	
781782	terminator	*241T>G	-	-	
781801	terminator	*260C>A	*260C>A	-	
781883	terminator	*342G>A	-	-	
781940	terminator	*399G>T	-	-	
782001	terminator	-	*460A>G	*460A>C	
782009	terminator	-	*468A>C	-	
782015	terminator	*474T>A	-	-	
782018	terminator	-	*477T>G	-	
782061	terminator	*520A>T	*520A>T	-	

Note: Positions with more than one variant change are shown in red. Previously known residues to modulate antifolate resistance are hightlighted in blue.

[+]Variants selected for validation
