# Supplementary material for: Reverse Chemical Genetics: Comprehensive Fitness Profiling Reveals the Spectrum of Drug Target Interactions
Source: PLoS Genet. 2016 Sep 2;12(9):e1006275. doi: 10.1371/journal.pgen.1006275 (PMC5010250; doi:10.1371/journal.pgen.1006275)
Supplement: S3 Table — (PDF) [file pgen.1006275.s009.pdf]

S3 Table. Variant calls in the haploid *dfr1*Δ pool.

Position	Region	T1	T2	T3	Residue
780475	promoter	-431T>C	-431T>C	-431T>C	
780516	promoter	-	-	-390G>A	
780585	promoter	-321A>G	-321A>G	-321A>G	
780612	promoter	-294T>A	-294T>A	-294T>A	
780619	promoter	-287A>T	-287A>T	-287A>T	
780657	promoter	-	-	-249A>T	
780666	promoter	-	-	-240G>A	
780756	promoter	-	-150G>A	-150G>A	
780803	promoter	-	-	-103G>A	
780819	promoter	-	-	-87A>G	
780833	promoter	-73C>T	-	-	
780852	promoter	-	-	-54C>T	
780903	promoter	-	-	-3A>T	
780953	cds	-	-	48G>A	Q16Q+
781122	cds	217A>T	-	-	N73Y+
781123	cds	218A>T	218A>T	218A>T	N73F
781155	cds	250A>G	250A>G	-	K84E+
781211	cds	306A>T	306A>T	306A>T	A102A
781217	cds	312A>G	312A>G	-	A104A+
781229	cds	324A>G	324A>G	324A>G	L108L
781253	cds	-	-	348G>A	L116L+
781284	cds	379G>A	-	-	V127I+
781328	cds	-	-	423G>A	T141T+
781331	cds	426A>G	426A>G	426A>G	K142K+
781358	cds	453T>A	453T>A	453T>A	T151T
781374	cds	-	-	469T>C	F157L+
781450	cds	-	-	545A>T	K182I
781481	cds	-	576A>T	576A>T	Q192H
781502	cds	-	-	597A>T	K199N
781532	cds	-	627T>C	-	N209N+
781547	terminator	*6C>T	-	-	
781572	terminator	*31T>G	*31T>G	-	
781620	terminator	-	-	*79C>T	
781622	terminator	*81A>G	-	-	
781633	terminator	*92T>A	-	-	
781673	terminator	*132G>A	*132G>A	*132G>A	
781728	terminator	*187T>A	*187T>A	*187T>A	
781749	terminator	-	-	*208G>A	
781774	terminator	-	*233C>T	-	
781819	terminator	-	*278T>A	-	
781825	terminator	-	-	*284C>T	
781910	terminator	*369T>A	-	-	
781955	terminator	*414T>A	*414T>A	-	
781974	terminator	-	-	*433C>T	
781996	terminator	*455G>T	*455G>T	-	
782028	terminator	-	-	*487C>T	
782049	terminator	*508C>A	-	-	
782060	terminator	*519G>A	*519G>A	*519G>A	
782068	terminator	*527G>A	*527G>A	*527G>A	

Note: Previously known residues to modulate antifolate resistance are hightlighted in blue.

+Variants selected for validation
