# Supplementary material for: Reverse Chemical Genetics: Comprehensive Fitness Profiling Reveals the Spectrum of Drug Target Interactions
Source: PLoS Genet. 2016 Sep 2;12(9):e1006275. doi: 10.1371/journal.pgen.1006275 (PMC5010250; doi:10.1371/journal.pgen.1006275)
Supplement: S4 Table — (PDF) [file pgen.1006275.s010.pdf]

S4 Table. The fitness profiles of *dfr1*, hDHFR and hDHFRL1 alleles in the diploid *DFR1*/*dfr1*Δ background.

Allele	Viability	Growth rate (hr)	Relative growth in MTX
DFR1/*dfr1*Δ	yes	6.6+/-0.0	62.1%+/-1.8%
DFR1/*dfr1*(I11V)	yes (p)	8.7+/-0.5	63.02%+/-4.5%
DFR1/*dfr1*(I11T)	yes	6.2+/-0.1	67.5%+/-2.9%
DFR1/*dfr1*(L15L)	yes	5.9+/-0.3	47.1%+/-1.6%
DFR1/*dfr1*(G25G)	yes	5.9+/-0.1	68.7%+/-1.6%
DFR1/*dfr1*(L27Q)	yes (p)	7.1+/-0.0	92.8%+/-5.6%
DFR1/*dfr1*(L27L)	yes	4.7+/-0.4	59.3%+/-3.9%
DFR1/*dfr1*(W29R)	yes (p)	5.4+/-0.2	60.1%+/-5.7%
DFR1/*dfr1*(M35V)	yes	6.4+/-0.1	105.3%+/-3.2%
DFR1/*dfr1*(M35T)	yes	5.3+/-0.3	107.6%+/-4.9%
DFR1/*dfr1*(K36E)	yes (p)	6.1+/-0.1	52.8%+/-2.0%
DFR1/*dfr1*(F38I)	yes (p)	4.8+/-0.2	54.4%+/-2.6%
DFR1/*dfr1*(F38Y)	yes	3.6+/-0.0	97.8%+/-0.8%
DFR1/*dfr1*(R39G)	yes	9.8+/-0.2	63.1%+/-6.1%
DFR1/*dfr1*(Q40P)	yes (p)	9.9+/-0.2	80.9%+/-4.1%
DFR1/*dfr1*(I55M)	yes (p)	5.1+/-0.2	71.3%+/-6.9%
DFR1/*dfr1*(S63A)	yes (p)	12.1+/-0.4	64.2%+/-0.1%
DFR1/*dfr1*(I64T)	yes (p)	5.2+/-0.0	66.8%+/-0.6%
DFR1/*dfr1*(I64M)	yes (p)	10.6+/-0.0	69.6%+/-0.6%
DFR1/*dfr1*(P65P)	yes	5.1+/-0.2	66.9%+/-7.0%
DFR1/*dfr1*(F68L)	yes	4.7+/-0.1	74.9%+/-2.3%
DFR1/*dfr1*(E109K)	yes	8.5+/-0.1	92.5%+/-2.5%
DFR1/*dfr1*(R118G)	yes	6.1+/-0.0	106.4%+/-2.8%
DFR1/*dfr1*(T141A)	yes (p)	6.1+/-0.2	89.4%+/-1.7%
DFR1/*dfr1*(D155H)	yes (p)	12.1+/-0.1	60.9%+/-3.4%
DFR1/*dfr1*(T156A)	yes (p)	3.9+/-0.5	75.7%+/-4.9%
DFR1/*dfr1*(F167L)	yes	9.0+/-0.4	65.1%+/-2.9%
DFR1/*dfr1*(K176T)	yes	5.9+/-0.1	113.8%+/-6.9%
DFR1/*dfr1*(F178F)	yes	5.9+/-0.3	70.2%+/-3.1%
DFR1/*dfr1*(E187K)	yes (p)	5.9+/-0.1	72.9%+/-2.1%
DFR1/*dfr1*(K199E)	yes	6.0+/-0.6	86.6%+/-7.9%
DFR1/*dfr1*(C202F)	yes (p)	5.1+/-0.0	73.6%+/-4.6%
DFR1/*dfr1*(N209H)	yes (p)	15.7+/-3.0	68.6+/-13.6%
DFR1/*dfr1*(N209N)	yes	11.4+/-0.6	49.3%+/-2.4%
DFR1/DHFR (W25R)	yes	5.1+/-0.8	99.3%+/-8.2%
DFR1/DHFRL1(R25W)	yes	5.1+/-0.3	80.2%+/-5.4%
DFR1/DHFRL1	yes	6.3+/-0.1	104.1%+/-1.5%

Note: The average growth in the presence of MTX (1 mM) was measured relative to DMSO treatment (see Methods for details). Control strains: wild-type homozygous diploid BY4743 and heterozygote *DFR1*/*dfr1*Δ were included. All growth assays were repeated in 3 independent experiments and standard error was calculated for each mutant. Mutant alleles that exhibited a “petite” phenotype due to growth defects in obligate respiratory conditions, are annotated with (p).
