# Supplementary material for: Reverse Chemical Genetics: Comprehensive Fitness Profiling Reveals the Spectrum of Drug Target Interactions
Source: PLoS Genet. 2016 Sep 2;12(9):e1006275. doi: 10.1371/journal.pgen.1006275 (PMC5010250; doi:10.1371/journal.pgen.1006275)
Supplement: S5 Table — (PDF) [file pgen.1006275.s011.pdf]

S5 Table. The fitness profiles of *dfr1* alleles identified in the haploid *dfr1*Δ background.

Allele	Viability	Growth rate (hr) in the:		Relative growth in MTX in the:	
		Haploid	Heterozygote	Haploid	Heterozygote
BY4741	yes	5.1+/-0.0	5.7+/-0.0*	57.6%+/-0.5%	35.1%+/-2.0%*
BY4742	yes	5.3+/-0.0	-	53.7%+/-1.6%	-
dfr1(Q16Q)	yes	4.8+/-0.0	12.5+/-0.0	55.7%+/-0.9%	46.5%+/-0.9%
dfr1(N73Y)	yes	13.6+/-2.1 (p)	6.1+/-0.1	99.1%+/-3.9%	44.8%+/-3.0%
dfr1(K84E)	yes	9.2+/-0.1	5.9+/-0.1	96.7%+/-2.6%	72.3%+/-5.9%
dfr1(A104A)	yes	6.0+/-0.0	9.3+/-0.2	92.9%+/-0.6%	81.0%+/-1.0%
dfr1(L116L)	yes	6.4+/-0.0	8.5+/-0.2	77.9%+/-0.2%	58.9%+/-1.5%
dfr1(V127I)	yes	8.6+/-0.3	4.1+/-0.0	78.7%+/-1.1%	60.9%+/-2.0%
dfr1(T141T)	yes	12.5+/-2.5	6.3+/-0.0	94.0%+/-4.0%	56.7%+/-1.1%
dfr1(K142K)	yes	6.8+/-0.1	10.0+/-0.1	88.6%+/-3.3%	57.5%+/-1.4%
dfr1(F157L)	yes	6.7+/-0.1 (p)	9.3+/-0.3	82.7%+/-0.7%	54.5%+/2.3%
dfr1(N209N)	yes	5.0+/-0.0	11.4+/-0.6	79.3%+/-0.6%	49.3%+/-2.4%

*wild-type (BY4743)

Note: The average growth in the presence of MTX (1 mM) was measured relative to DMSO treatment (see Methods for details). Control strains: wild-type homozygous diploid BY4743, haploid BY4741 and BY4742 were included. All growth assays were repeated in 3 independent experiments and standard error was calculated for each mutant. Mutant alleles that exhibited a “petite” phenotype due to growth defects in obligate respiratory conditions, are annotated with (p).
