# Supplementary material for: Reverse Chemical Genetics: Comprehensive Fitness Profiling Reveals the Spectrum of Drug Target Interactions
Source: PLoS Genet. 2016 Sep 2;12(9):e1006275. doi: 10.1371/journal.pgen.1006275 (PMC5010250; doi:10.1371/journal.pgen.1006275)
Supplement: S6 Table — (PDF) [file pgen.1006275.s012.pdf]

S6 Table. Synthetic gene fragments used to reconstruct dfr1 mutants.

```
> Human DHFR(WT)
ACATTATGCTTTGCATGATAATAAGAGAAATTGAAGAGCGCAACGAACTACGAGCATGGTTGGTTCCTTGAACTGTATCGTCGCTGTCTCTCAAAACATGGGTATTGGTAAGAACGGTGACTTGCCATGGCCACCATTGAGAAACGAATTCAGAT
ATTTCCAAAGAATGACTACCACTTCCTCTGTCGAAGGTAAGCAAAACTTAGTTATTATGGGTAAGAAGACCTGGTTCTCTATTCCAGAGAAAAACAGACCATTGAAGGGTAGAATCAACTTGGTCTTGTCTAGAGAGTTAAAGGAACCACCTCAA
GGTGCCCATTTCTTGTCCAGATCCTTGGATGATGCTTTGAAGTTGACTGAACAACCAGAATTGGCTAACAAGGTTGACATGGTTTGGATCGTTGGTGGTTCTTCCGTTTACAAGGAAGCCATGAACCATCCAGGTCATTTGAAGTTGTTCGTCAC
TAGAATCATGCAAGACTTCGAATCTGATACTTTTTTCCCTGAAATCGATTTAGAAAAATACAAATTGTTACCAGAATACCCAGGTGTTTTATCTGATGTCCAAGAAGAAAAGGGTATCAAGTACAAATTCGAGGTTTATGAAAAGAACGATAACC
TCTCCGCCCG

> Human DHFR(W25R)
ACATTATGCTTTGCATGATAATAAGAGAAATTGAAGAGCGCAACGAACTACGAGCATGGTTGGTTCCTTGAACTGTATCGTCGCTGTCTCTCAAAACATGGGTATTGGTAAGAACGGTGACTTGCCACGGCCACCATTGAGAAACGAATTCAGAT
ATTTCCAAAGAATGACTACCACTTCCTCTGTCGAAGGTAAGCAAAACTTAGTTATTATGGGTAAGAAGACCTGGTTCTCTATTCCAGAGAAAAACAGACCATTGAAGGGTAGAATCAACTTGGTCTTGTCTAGAGAGTTAAAGGAACCACCTCAA
GGTGCCCATTTCTTGTCCAGATCCTTGGATGATGCTTTGAAGTTGACTGAACAACCAGAATTGGCTAACAAGGTTGACATGGTTTGGATCGTTGGTGGTTCTTCCGTTTACAAGGAAGCCATGAACCATCCAGGTCATTTGAAGTTGTTCGTCAC
TAGAATCATGCAAGACTTCGAATCTGATACTTTTTTCCCTGAAATCGATTTAGAAAAATACAAATTGTTACCAGAATACCCAGGTGTTTTATCTGATGTCCAAGAAGAAAAGGGTATCAAGTACAAATTCGAGGTTTATGAAAAGAACGATAACC
TCTCCGCCCG

> Human DHFRL1 (WT)
ACATTATGCTTTGCATGATAATAAGAGAAATTGAAGAGCGCAACGAACTACGAGCATGTTCTTGTTATTGAACTGTATCGTTGCTGTCTCCCAAAACATGGGTATTGGTAAGAATGGTGATTTGCCACGTCCACCATTGAGAAATGAATTCAGAT
ATTTCCAACGTATGACCACCACCTCTTCTGTTGAGGGTAAACAAAACTTAGTCATTATGGGTAGAAAGACTTGGTTCTCTATTCCTGAAAAGAACCGTCCATTAAAGGATAGAATCAACTTGGTCTTGTCTAGAGAATTGAAAGAGCCACCTCAA
GGTGCTCACTTCTTGGCTCGTTCTTTGGACGATGCCTTAAAGTTAACTGAAAGACCAGAATTGGCTAATAAGGTTGACATGATCTGGATTGTTGGTGGTTCTTCTGTCTATAAGGAAGCTATGAACCACTTGGGTCACTTGAAGTTATTCGTTAC
TAGAATCATGCAAGACTTTGAATCTGATACTTTCTTTTCTGAAATCGACTTGGAAAAGTACAAGTTGTTGCCAGAGTACCCTGGTGTTTTGTCTGACGTTCAAGAAGGTAAGCACATCAAGTACAAATTCGAAGTTTGTGAAAAGGACGACAACC
TCTCCGCCCG

> Human DHFRL1 (R25W)
ACATTATGCTTTGCATGATAATAAGAGAAATTGAAGAGCGCAACGAACTACGAGCATGTTCTTGTTATTGAACTGTATCGTTGCTGTCTCCCAAAACATGGGTATTGGTAAGAATGGTGATTTGCCATGGCCACCATTGAGAAATGAATTCAGAT
ATTTCCAACGTATGACCACCACCTCTTCTGTTGAGGGTAAACAAAACTTAGTCATTATGGGTAGAAAGACTTGGTTCTCTATTCCTGAAAAGAACCGTCCATTAAAGGATAGAATCAACTTGGTCTTGTCTAGAGAATTGAAAGAGCCACCTCAA
GGTGCTCACTTCTTGGCTCGTTCTTTGGACGATGCCTTAAAGTTAACTGAAAGACCAGAATTGGCTAATAAGGTTGACATGATCTGGATTGTTGGTGGTTCTTCTGTCTATAAGGAAGCTATGAACCACTTGGGTCACTTGAAGTTATTCGTTAC
TAGAATCATGCAAGACTTTGAATCTGATACTTTCTTTTCTGAAATCGACTTGGAAAAGTACAAGTTGTTGCCAGAGTACCCTGGTGTTTTGTCTGACGTTCAAGAAGGTAAGCACATCAAGTACAAATTCGAAGTTTGTGAAAAGGACGACAACC
TCTCCGCCCG

> DFR1(V127I)
ACATTATGCTTTGCATGATAATAAGAGAAATTGAAGAGCGCAACGAACTACGAGCATGGCTGGAGGAAAGATTCCTATTGTAGGAATTGTGGCATGTTTACAGCCGGAGATGGGGATAGGATTTCGTGGAGGTCTACCATGGAGGTTGCCCAGTG
AAATGAAGTATTTCAGACAGGTCACTTCATTGACGAAAGATCCAAACAAAAAAAATGCTTTGATAATGGGAAGGAAGACATGGGAATCCATACCGCCCAAGTTTCGCCCACTGCCCAATAGAATGAATGTCATTATATCGAGAAGCTTCAAGGAC
GATTTTGTCCACGATAAAGAGAGATCAATAGTCCAAAGTAATTCATTGGCAAACGCAATAATGAACCTAGAAAGCAATTTTAAGGAGCATCTGGAAAGAATCTACGTGATTGGGGGTGGCGAAATTTATAGTCAAATCTTCTCCATTACAGATCA
TTGGCTCATCACGAAAATAAATCCATTAGATAAAAACGCAACTCCTGCAATGGACACTTTCCTTGATGCGAAGAAATTGGAAGAAGTATTTAGCGAGCAAGATCCGGCCCAGCTGAAAGAATTTCTTCCCCCTAAAGTAGAGTTGCCCGAAACAG
ACTGTGATCAACGCTACTCGCTGGAAGAAAAAGGTTATTGCTTCGAATTCACTCTATACAATCGTAAATGAAACCTCTCCGCCCG

> DFR1(F157L)
ACATTATGCTTTGCATGATAATAAGAGAAATTGAAGAGCGCAACGAACTACGAGCATGGCTGGAGGAAAGATTCCTATTGTAGGAATTGTGGCATGTTTACAGCCGGAGATGGGGATAGGATTTCGTGGAGGTCTACCATGGAGGTTGCCCAGTG
AAATGAAGTATTTCAGACAGGTCACTTCATTGACGAAAGATCCAAACAAAAAAAATGCTTTGATAATGGGAAGGAAGACATGGGAATCCATACCGCCCAAGTTTCGCCCACTGCCCAATAGAATGAATGTCATTATATCGAGAAGCTTCAAGGAC
GATTTTGTCCACGATAAAGAGAGATCAATAGTCCAAAGTAATTCATTGGCAAACGCAATAATGAACCTAGAAAGCAATTTTAAGGAGCATCTGGAAAGAATCTACGTGATTGGGGGTGGCGAAGTTTATAGTCAAATCTTCTCCATTACAGATCA
TTGGCTCATCACGAAAATAAATCCATTAGATAAAAACGCAACTCCTGCAATGGACACTCTCCTTGATGCGAAGAAATTGGAAGAAGTATTTAGCGAGCAAGATCCGGCCCAGCTGAAAGAATTTCTTCCCCCTAAAGTAGAGTTGCCCGAAACAG
ACTGTGATCAACGCTACTCGCTGGAAGAAAAAGGTTATTGCTTCGAATTCACTCTATACAATCGTAAATGAAACCTCTCCGCCCG

> DFR1(K84E)
ACATTATGCTTTGCATGATAATAAGAGAAATTGAAGAGCGCAACGAACTACGAGCATGGCTGGAGGAAAGATTCCTATTGTAGGAATTGTGGCATGTTTACAGCCGGAGATGGGGATAGGATTTCGTGGAGGTCTACCATGGAGGTTGCCCAGTG
AAATGAAGTATTTCAGACAGGTCACTTCATTGACGAAAGATCCAAACAAAAAAAATGCTTTGATAATGGGAAGGAAGACATGGGAATCCATACCGCCCAAGTTTCGCCCACTGCCCAATAGAATGAATGTCATTATATCGAGAAGCTTCGAGGAC
GATTTTGTCCACGATAAAGAGAGATCAATAGTCCAAAGTAATTCATTGGCAAACGCAATAATGAACCTAGAAAGCAATTTTAAGGAGCATCTGGAAAGAATCTACGTGATTGGGGGTGGCGAAGTTTATAGTCAAATCTTCTCCATTACAGATCA
TTGGCTCATCACGAAAATAAATCCATTAGATAAAAACGCAACTCCTGCAATGGACACTTTCCTTGATGCGAAGAAATTGGAAGAAGTATTTAGCGAGCAAGATCCGGCCCAGCTGAAAGAATTTCTTCCCCCTAAAGTAGAGTTGCCCGAAACAG
ACTGTGATCAACGCTACTCGCTGGAAGAAAAAGGTTATTGCTTCGAATTCACTCTATACAATCGTAAATGAAACCTCTCCGCCCG

> DFR1(N73Y)
ACATTATGCTTTGCATGATAATAAGAGAAATTGAAGAGCGCAACGAACTACGAGCATGGCTGGAGGAAAGATTCCTATTGTAGGAATTGTGGCATGTTTACAGCCGGAGATGGGGATAGGATTTCGTGGAGGTCTACCATGGAGGTTGCCCAGTG
AAATGAAGTATTTCAGACAGGTCACTTCATTGACGAAAGATCCAAACAAAAAAAATGCTTTGATAATGGGAAGGAAGACATGGGAATCCATACCGCCCAAGTTTCGCCCACTGCCCTATAGAATGAATGTCATTATATCGAGAAGCTTCAAGGAC
GATTTTGTCCACGATAAAGAGAGATCAATAGTCCAAAGTAATTCATTGGCAAACGCAATAATGAACCTAGAAAGCAATTTTAAGGAGCATCTGGAAAGAATCTACGTGATTGGGGGTGGCGAAGTTTATAGTCAAATCTTCTCCATTACAGATCA
TTGGCTCATCACGAAAATAAATCCATTAGATAAAAACGCAACTCCTGCAATGGACACTTTCCTTGATGCGAAGAAATTGGAAGAAGTATTTAGCGAGCAAGATCCGGCCCAGCTGAAAGAATTTCTTCCCCCTAAAGTAGAGTTGCCCGAAACAG
ACTGTGATCAACGCTACTCGCTGGAAGAAAAAGGTTATTGCTTCGAATTCACTCTATACAATCGTAAATGAAACCTCTCCGCCCG

> DFR1(A104A)
ACATTATGCTTTGCATGATAATAAGAGAAATTGAAGAGCGCAACGAACTACGAGCATGGCTGGAGGAAAGATTCCTATTGTAGGAATTGTGGCATGTTTACAGCCGGAGATGGGGATAGGATTTCGTGGAGGTCTACCATGGAGGTTGCCCAGTG
AAATGAAGTATTTCAGACAGGTCACTTCATTGACGAAAGATCCAAACAAAAAAAATGCTTTGATAATGGGAAGGAAGACATGGGAATCCATACCGCCCAAGTTTCGCCCACTGCCCAATAGAATGAATGTCATTATATCGAGAAGCTTCAAGGAC
GATTTTGTCCACGATAAAGAGAGATCAATAGTCCAAAGTAATTCATTGGCAAACGCGATAATGAACCTAGAAAGCAATTTTAAGGAGCATCTGGAAAGAATCTACGTGATTGGGGGTGGCGAAGTTTATAGTCAAATCTTCTCCATTACAGATCA
TTGGCTCATCACGAAAATAAATCCATTAGATAAAAACGCAACTCCTGCAATGGACACTTTCCTTGATGCGAAGAAATTGGAAGAAGTATTTAGCGAGCAAGATCCGGCCCAGCTGAAAGAATTTCTTCCCCCTAAAGTAGAGTTGCCCGAAACAG
ACTGTGATCAACGCTACTCGCTGGAAGAAAAAGGTTATTGCTTCGAATTCACTCTATACAATCGTAAATGAAACCTCTCCGCCCG

> DFR1(Q16Q)
ACATTATGCTTTGCATGATAATAAGAGAAATTGAAGAGCGCAACGAACTACGAGCATGGCTGGAGGAAAGATTCCTATTGTAGGAATTGTGGCATGTTTACAACCGGAGATGGGGATAGGATTTCGTGGAGGTCTACCATGGAGGTTGCCCAGTG
AAATGAAGTATTTCAGACAGGTCACTTCATTGACGAAAGATCCAAACAAAAAAAATGCTTTGATAATGGGAAGGAAGACATGGGAATCCATACCGCCCAAGTTTCGCCCACTGCCCAATAGAATGAATGTCATTATATCGAGAAGCTTCAAGGAC
GATTTTGTCCACGATAAAGAGAGATCAATAGTCCAAAGTAATTCATTGGCAAACGCAATAATGAACCTAGAAAGCAATTTTAAGGAGCATCTGGAAAGAATCTACGTGATTGGGGGTGGCGAAGTTTATAGTCAAATCTTCTCCATTACAGATCA
TTGGCTCATCACGAAAATAAATCCATTAGATAAAAACGCAACTCCTGCAATGGACACTTTCCTTGATGCGAAGAAATTGGAAGAAGTATTTAGCGAGCAAGATCCGGCCCAGCTGAAAGAATTTCTTCCCCCTAAAGTAGAGTTGCCCGAAACAG
ACTGTGATCAACGCTACTCGCTGGAAGAAAAAGGTTATTGCTTCGAATTCACTCTATACAATCGTAAATGAAACCTCTCCGCCCG

> DFR1(K142K)
ACATTATGCTTTGCATGATAATAAGAGAAATTGAAGAGCGCAACGAACTACGAGCATGGCTGGAGGAAAGATTCCTATTGTAGGAATTGTGGCATGTTTACAGCCGGAGATGGGGATAGGATTTCGTGGAGGTCTACCATGGAGGTTGCCCAGTG
AAATGAAGTATTTCAGACAGGTCACTTCATTGACGAAAGATCCAAACAAAAAAAATGCTTTGATAATGGGAAGGAAGACATGGGAATCCATACCGCCCAAGTTTCGCCCACTGCCCAATAGAATGAATGTCATTATATCGAGAAGCTTCAAGGAC
GATTTTGTCCACGATAAAGAGAGATCAATAGTCCAAAGTAATTCATTGGCAAACGCAATAATGAACCTAGAAAGCAATTTTAAGGAGCATCTGGAAAGAATCTACGTGATTGGGGGTGGCGAAGTTTATAGTCAAATCTTCTCCATTACAGATCA
TTGGCTCATCACGAAGATAAATCCATTAGATAAAAACGCAACTCCTGCAATGGACACTTTCCTTGATGCGAAGAAATTGGAAGAAGTATTTAGCGAGCAAGATCCGGCCCAGCTGAAAGAATTTCTTCCCCCTAAAGTAGAGTTGCCCGAAACAG
ACTGTGATCAACGCTACTCGCTGGAAGAAAAAGGTTATTGCTTCGAATTCACTCTATACAATCGTAAATGAAACCTCTCCGCCCG

> DFR1(T141T)
ACATTATGCTTTGCATGATAATAAGAGAAATTGAAGAGCGCAACGAACTACGAGCATGGCTGGAGGAAAGATTCCTATTGTAGGAATTGTGGCATGTTTACAGCCGGAGATGGGGATAGGATTTCGTGGAGGTCTACCATGGAGGTTGCCCAGTG
AAATGAAGTATTTCAGACAGGTCACTTCATTGACGAAAGATCCAAACAAAAAAAATGCTTTGATAATGGGAAGGAAGACATGGGAATCCATACCGCCCAAGTTTCGCCCACTGCCCAATAGAATGAATGTCATTATATCGAGAAGCTTCAAGGAC
GATTTTGTCCACGATAAAGAGAGATCAATAGTCCAAAGTAATTCATTGGCAAACGCAATAATGAACCTAGAAAGCAATTTTAAGGAGCATCTGGAAAGAATCTACGTGATTGGGGGTGGCGAAGTTTATAGTCAAATCTTCTCCATTACAGATCA
TTGGCTCATCACAAAAATAAATCCATTAGATAAAAACGCAACTCCTGCAATGGACACTTTCCTTGATGCGAAGAAATTGGAAGAAGTATTTAGCGAGCAAGATCCGGCCCAGCTGAAAGAATTTCTTCCCCCTAAAGTAGAGTTGCCCGAAACAG
ACTGTGATCAACGCTACTCGCTGGAAGAAAAAGGTTATTGCTTCGAATTCACTCTATACAATCGTAAATGAAACCTCTCCGCCCG

> DFR1(N209N)
ACATTATGCTTTGCATGATAATAAGAGAAATTGAAGAGCGCAACGAACTACGAGCATGGCTGGAGGAAAGATTCCTATTGTAGGAATTGTGGCATGTTTACAGCCGGAGATGGGGATAGGATTTCGTGGAGGTCTACCATGGAGGTTGCCCAGTG
AAATGAAGTATTTCAGACAGGTCACTTCATTGACGAAAGATCCAAACAAAAAAAATGCTTTGATAATGGGAAGGAAGACATGGGAATCCATACCGCCCAAGTTTCGCCCACTGCCCAATAGAATGAATGTCATTATATCGAGAAGCTTCAAGGAC
GATTTTGTCCACGATAAAGAGAGATCAATAGTCCAAAGTAATTCATTGGCAAACGCAATAATGAACCTAGAAAGCAATTTTAAGGAGCATCTGGAAAGAATCTACGTGATTGGGGGTGGCGAAGTTTATAGTCAAATCTTCTCCATTACAGATCA
TTGGCTCATCACGAAAATAAATCCATTAGATAAAAACGCAACTCCTGCAATGGACACTTTCCTTGATGCGAAGAAATTGGAAGAAGTATTTAGCGAGCAAGATCCGGCCCAGCTGAAAGAATTTCTTCCCCCTAAAGTAGAGTTGCCCGAAACAG
ACTGTGATCAACGCTACTCGCTGGAAGAAAAAGGTTATTGCTTCGAATTCACTCTATACAACCGTAAATGAAACCTCTCCGCCCG

> DFR1(L116L)
ACATTATGCTTTGCATGATAATAAGAGAAATTGAAGAGCGCAACGAACTACGAGCATGGCTGGAGGAAAGATTCCTATTGTAGGAATTGTGGCATGTTTACAGCCGGAGATGGGGATAGGATTTCGTGGAGGTCTACCATGGAGGTTGCCCAGTG
AAATGAAGTATTTCAGACAGGTCACTTCATTGACGAAAGATCCAAACAAAAAAAATGCTTTGATAATGGGAAGGAAGACATGGGAATCCATACCGCCCAAGTTTCGCCCACTGCCCAATAGAATGAATGTCATTATATCGAGAAGCTTCAAGGAC
GATTTTGTCCACGATAAAGAGAGATCAATAGTCCAAAGTAATTCATTGGCAAACGCAATAATGAACCTAGAAAGCAATTTTAAGGAGCATCTAGAAAGAATCTACGTGATTGGGGGTGGCGAAGTTTATAGTCAAATCTTCTCCATTACAGATCA
TTGGCTCATCACGAAAATAAATCCATTAGATAAAAACGCAACTCCTGCAATGGACACTTTCCTTGATGCGAAGAAATTGGAAGAAGTATTTAGCGAGCAAGATCCGGCCCAGCTGAAAGAATTTCTTCCCCCTAAAGTAGAGTTGCCCGAAACAG
ACTGTGATCAACGCTACTCGCTGGAAGAAAAAGGTTATTGCTTCGAATTCACTCTATACAATCGTAAATGAAACCTCTCCGCCCG

> DFR1 (I11V)
ACATTATGCTTTGCATGATAATAAGAGAAATTGAAGAGCGCAACGAACTACGAGCATGGCTGGAGGAAAGATTCCTATTGTAGGAGTTGTGGCATGTTTACAGCCGGAGATGGGGATAGGATTTCGTGGAGGTCTACCATGGAGGTTGCCCAGTG
AAATGAAGTATTTCAGACAGGTCACTTCATTGACGAAAGATCCAAACAAAAAAAATGCTTTGATAATGGGAAGGAAGACATGGGAATCCATACCGCCCAAGTTTCGCCCACTGCCCAATAGAATGAATGTCATTATATCGAGAAGCTTCAAGGAC
GATTTTGTCCACGATAAAGAGAGATCAATAGTCCAAAGTAATTCATTGGCAAACGCAATAATGAACCTAGAAAGCAATTTTAAGGAGCATCTGGAAAGAATCTACGTGATTGGGGGTGGCGAAGTTTATAGTCAAATCTTCTCCATTACAGATCA
TTGGCTCATCACGAAAATAAATCCATTAGATAAAAACGCAACTCCTGCAATGGACACTTTCCTTGATGCGAAGAAATTGGAAGAAGTATTTAGCGAGCAAGATCCGGCCCAGCTGAAAGAATTTCTTCCCCCTAAAGTAGAGTTGCCCGAAACAG
ACTGTGATCAACGCTACTCGCTGGAAGAAAAAGGTTATTGCTTCGAATTCACTCTATACAATCGTAAATGAAACCTCTCCGCCCG

> DFR1 (I11T)
ACATTATGCTTTGCATGATAATAAGAGAAATTGAAGAGCGCAACGAACTACGAGCATGGCTGGAGGAAAGATTCCTATTGTAGGAACTGTGGCATGTTTACAGCCGGAGATGGGGATAGGATTTCGTGGAGGTCTACCATGGAGGTTGCCCAGTG
AAATGAAGTATTTCAGACAGGTCACTTCATTGACGAAAGATCCAAACAAAAAAAATGCTTTGATAATGGGAAGGAAGACATGGGAATCCATACCGCCCAAGTTTCGCCCACTGCCCAATAGAATGAATGTCATTATATCGAGAAGCTTCAAGGAC
```

GATTTTGTCCACGATAAAGAGAGATCAATAGTCCAAAGTAATTCATTGGCAAACGCAATAATGAACCTAGAAAGCAATTTTAAGGAGCATCTGGAAAGAATCTACGTGATTGGGGGTGGCGAAGTTTATAGTCAAATCTTCTCCATTACAGATCA
TTGGCTCATCACGAAAATAAATCCATTAGATAAAAACGCAACTCCTGCAATGGACACTTTCCTTGATGCGAAGAAATTGGAAGAAGTATTTAGCGAGCAAGATCCGGCCCAGCTGAAAGAATTTCTTCCCCCTAAAGTAGAGTTGCCCGAAACAG
ACTGTGATCAACGCTACTCGCTGGAAGAAAAAGGTTATTGCTTCGAATTCACTCTATACAATCGTAAATGAAACCTCTCCGCCCG

> DFR1 (L15L)
ACATTATGCTTTGCATGATAATAAGAGAAATTGAAGAGCGCAACGAACTACGAGCATGGCTGGAGGAAAGATTCCTATTGTAGGAATTGTGGCATGTTTGCAGCCGGAGATGGGGATAGGATTTCGTGGAGGTCTACCATGGAGGTTGCCCAGTG
AAATGAAGTATTTCAGACAGGTCACTTCATTGACGAAAGATCCAAACAAAAAAAATGCTTTGATAATGGGAAGGAAGACATGGGAATCCATACCGCCCAAGTTTCGCCCACTGCCCAATAGAATGAATGTCATTATATCGAGAAGCTTCAAGGAC
GATTTTGTCCACGATAAAGAGAGATCAATAGTCCAAAGTAATTCATTGGCAAACGCAATAATGAACCTAGAAAGCAATTTTAAGGAGCATCTGGAAAGAATCTACGTGATTGGGGGTGGCGAAGTTTATAGTCAAATCTTCTCCATTACAGATCA
TTGGCTCATCACGAAAATAAATCCATTAGATAAAAACGCAACTCCTGCAATGGACACTTTCCTTGATGCGAAGAAATTGGAAGAAGTATTTAGCGAGCAAGATCCGGCCCAGCTGAAAGAATTTCTTCCCCCTAAAGTAGAGTTGCCCGAAACAG
ACTGTGATCAACGCTACTCGCTGGAAGAAAAAGGTTATTGCTTCGAATTCACTCTATACAATCGTAAATGAAACCTCTCCGCCCG

> DFR1 (G25G)
ACATTATGCTTTGCATGATAATAAGAGAAATTGAAGAGCGCAACGAACTACGAGCATGGCTGGAGGAAAGATTCCTATTGTAGGAATTGTGGCATGTTTACAGCCGGAGATGGGGATAGGATTTCGTGGGGGTCTACCATGGAGGTTGCCCAGTG
AAATGAAGTATTTCAGACAGGTCACTTCATTGACGAAAGATCCAAACAAAAAAAATGCTTTGATAATGGGAAGGAAGACATGGGAATCCATACCGCCCAAGTTTCGCCCACTGCCCAATAGAATGAATGTCATTATATCGAGAAGCTTCAAGGAC
GATTTTGTCCACGATAAAGAGAGATCAATAGTCCAAAGTAATTCATTGGCAAACGCAATAATGAACCTAGAAAGCAATTTTAAGGAGCATCTGGAAAGAATCTACGTGATTGGGGGTGGCGAAGTTTATAGTCAAATCTTCTCCATTACAGATCA
TTGGCTCATCACGAAAATAAATCCATTAGATAAAAACGCAACTCCTGCAATGGACACTTTCCTTGATGCGAAGAAATTGGAAGAAGTATTTAGCGAGCAAGATCCGGCCCAGCTGAAAGAATTTCTTCCCCCTAAAGTAGAGTTGCCCGAAACAG
ACTGTGATCAACGCTACTCGCTGGAAGAAAAAGGTTATTGCTTCGAATTCACTCTATACAATCGTAAATGAAACCTCTCCGCCCG

> DFR1 (L27Q)
ACATTATGCTTTGCATGATAATAAGAGAAATTGAAGAGCGCAACGAACTACGAGCATGGCTGGAGGAAAGATTCCTATTGTAGGAATTGTGGCATGTTTACAGCCGGAGATGGGGATAGGATTTCGTGGAGGTCAACCATGGAGGTTGCCCAGTG
AAATGAAGTATTTCAGACAGGTCACTTCATTGACGAAAGATCCAAACAAAAAAAATGCTTTGATAATGGGAAGGAAGACATGGGAATCCATACCGCCCAAGTTTCGCCCACTGCCCAATAGAATGAATGTCATTATATCGAGAAGCTTCAAGGAC
GATTTTGTCCACGATAAAGAGAGATCAATAGTCCAAAGTAATTCATTGGCAAACGCAATAATGAACCTAGAAAGCAATTTTAAGGAGCATCTGGAAAGAATCTACGTGATTGGGGGTGGCGAAGTTTATAGTCAAATCTTCTCCATTACAGATCA
TTGGCTCATCACGAAAATAAATCCATTAGATAAAAACGCAACTCCTGCAATGGACACTTTCCTTGATGCGAAGAAATTGGAAGAAGTATTTAGCGAGCAAGATCCGGCCCAGCTGAAAGAATTTCTTCCCCCTAAAGTAGAGTTGCCCGAAACAG
ACTGTGATCAACGCTACTCGCTGGAAGAAAAAGGTTATTGCTTCGAATTCACTCTATACAATCGTAAATGAAACCTCTCCGCCCG

> DFR1 (L27L)
ACATTATGCTTTGCATGATAATAAGAGAAATTGAAGAGCGCAACGAACTACGAGCATGGCTGGAGGAAAGATTCCTATTGTAGGAATTGTGGCATGTTTACAGCCGGAGATGGGGATAGGATTTCGTGGAGGTCTGCCATGGAGGTTGCCCAGTG
AAATGAAGTATTTCAGACAGGTCACTTCATTGACGAAAGATCCAAACAAAAAAAATGCTTTGATAATGGGAAGGAAGACATGGGAATCCATACCGCCCAAGTTTCGCCCACTGCCCAATAGAATGAATGTCATTATATCGAGAAGCTTCAAGGAC
GATTTTGTCCACGATAAAGAGAGATCAATAGTCCAAAGTAATTCATTGGCAAACGCAATAATGAACCTAGAAAGCAATTTTAAGGAGCATCTGGAAAGAATCTACGTGATTGGGGGTGGCGAAGTTTATAGTCAAATCTTCTCCATTACAGATCA
TTGGCTCATCACGAAAATAAATCCATTAGATAAAAACGCAACTCCTGCAATGGACACTTTCCTTGATGCGAAGAAATTGGAAGAAGTATTTAGCGAGCAAGATCCGGCCCAGCTGAAAGAATTTCTTCCCCCTAAAGTAGAGTTGCCCGAAACAG
ACTGTGATCAACGCTACTCGCTGGAAGAAAAAGGTTATTGCTTCGAATTCACTCTATACAATCGTAAATGAAACCTCTCCGCCCG

> DFR1 (W29R)
ACATTATGCTTTGCATGATAATAAGAGAAATTGAAGAGCGCAACGAACTACGAGCATGGCTGGAGGAAAGATTCCTATTGTAGGAATTGTGGCATGTTTACAGCCGGAGATGGGGATAGGATTTCGTGGAGGTCTACCACGGAGGTTGCCCAGTG
AAATGAAGTATTTCAGACAGGTCACTTCATTGACGAAAGATCCAAACAAAAAAAATGCTTTGATAATGGGAAGGAAGACATGGGAATCCATACCGCCCAAGTTTCGCCCACTGCCCAATAGAATGAATGTCATTATATCGAGAAGCTTCAAGGAC
GATTTTGTCCACGATAAAGAGAGATCAATAGTCCAAAGTAATTCATTGGCAAACGCAATAATGAACCTAGAAAGCAATTTTAAGGAGCATCTGGAAAGAATCTACGTGATTGGGGGTGGCGAAGTTTATAGTCAAATCTTCTCCATTACAGATCA
TTGGCTCATCACGAAAATAAATCCATTAGATAAAAACGCAACTCCTGCAATGGACACTTTCCTTGATGCGAAGAAATTGGAAGAAGTATTTAGCGAGCAAGATCCGGCCCAGCTGAAAGAATTTCTTCCCCCTAAAGTAGAGTTGCCCGAAACAG
ACTGTGATCAACGCTACTCGCTGGAAGAAAAAGGTTATTGCTTCGAATTCACTCTATACAATCGTAAATGAAACCTCTCCGCCCG

> DFR1 (M35V)
ACATTATGCTTTGCATGATAATAAGAGAAATTGAAGAGCGCAACGAACTACGAGCATGGCTGGAGGAAAGATTCCTATTGTAGGAATTGTGGCATGTTTACAGCCGGAGATGGGGATAGGATTTCGTGGAGGTCTACCATGGAGGTTGCCCAGTG
AAGTGAAGTATTTCAGACAGGTCACTTCATTGACGAAAGATCCAAACAAAAAAAATGCTTTGATAATGGGAAGGAAGACATGGGAATCCATACCGCCCAAGTTTCGCCCACTGCCCAATAGAATGAATGTCATTATATCGAGAAGCTTCAAGGAC
GATTTTGTCCACGATAAAGAGAGATCAATAGTCCAAAGTAATTCATTGGCAAACGCAATAATGAACCTAGAAAGCAATTTTAAGGAGCATCTGGAAAGAATCTACGTGATTGGGGGTGGCGAAGTTTATAGTCAAATCTTCTCCATTACAGATCA
TTGGCTCATCACGAAAATAAATCCATTAGATAAAAACGCAACTCCTGCAATGGACACTTTCCTTGATGCGAAGAAATTGGAAGAAGTATTTAGCGAGCAAGATCCGGCCCAGCTGAAAGAATTTCTTCCCCCTAAAGTAGAGTTGCCCGAAACAG
ACTGTGATCAACGCTACTCGCTGGAAGAAAAAGGTTATTGCTTCGAATTCACTCTATACAATCGTAAATGAAACCTCTCCGCCCG

> DFR1 (M35T)
ACATTATGCTTTGCATGATAATAAGAGAAATTGAAGAGCGCAACGAACTACGAGCATGGCTGGAGGAAAGATTCCTATTGTAGGAATTGTGGCATGTTTACAGCCGGAGATGGGGATAGGATTTCGTGGAGGTCTACCATGGAGGTTGCCCAGTG
AAACGAAGTATTTCAGACAGGTCACTTCATTGACGAAAGATCCAAACAAAAAAAATGCTTTGATAATGGGAAGGAAGACATGGGAATCCATACCGCCCAAGTTTCGCCCACTGCCCAATAGAATGAATGTCATTATATCGAGAAGCTTCAAGGAC
GATTTTGTCCACGATAAAGAGAGATCAATAGTCCAAAGTAATTCATTGGCAAACGCAATAATGAACCTAGAAAGCAATTTTAAGGAGCATCTGGAAAGAATCTACGTGATTGGGGGTGGCGAAGTTTATAGTCAAATCTTCTCCATTACAGATCA
TTGGCTCATCACGAAAATAAATCCATTAGATAAAAACGCAACTCCTGCAATGGACACTTTCCTTGATGCGAAGAAATTGGAAGAAGTATTTAGCGAGCAAGATCCGGCCCAGCTGAAAGAATTTCTTCCCCCTAAAGTAGAGTTGCCCGAAACAG
ACTGTGATCAACGCTACTCGCTGGAAGAAAAAGGTTATTGCTTCGAATTCACTCTATACAATCGTAAATGAAACCTCTCCGCCCG

> DFR1 (K36E)
ACATTATGCTTTGCATGATAATAAGAGAAATTGAAGAGCGCAACGAACTACGAGCATGGCTGGAGGAAAGATTCCTATTGTAGGAATTGTGGCATGTTTACAGCCGGAGATGGGGATAGGATTTCGTGGAGGTCTACCATGGAGGTTGCCCAGTG
AAACGGAGTATTTCAGACAGGTCACTTCATTGACGAAAGATCCAAACAAAAAAAATGCTTTGATAATGGGAAGGAAGACATGGGAATCCATACCGCCCAAGTTTCGCCCACTGCCCAATAGAATGAATGTCATTATATCGAGAAGCTTCAAGGAC
GATTTTGTCCACGATAAAGAGAGATCAATAGTCCAAAGTAATTCATTGGCAAACGCAATAATGAACCTAGAAAGCAATTTTAAGGAGCATCTGGAAAGAATCTACGTGATTGGGGGTGGCGAAGTTTATAGTCAAATCTTCTCCATTACAGATCA
TTGGCTCATCACGAAAATAAATCCATTAGATAAAAACGCAACTCCTGCAATGGACACTTTCCTTGATGCGAAGAAATTGGAAGAAGTATTTAGCGAGCAAGATCCGGCCCAGCTGAAAGAATTTCTTCCCCCTAAAGTAGAGTTGCCCGAAACAG
ACTGTGATCAACGCTACTCGCTGGAAGAAAAAGGTTATTGCTTCGAATTCACTCTATACAATCGTAAATGAAACCTCTCCGCCCG

> DFR1 (F38I)
ACATTATGCTTTGCATGATAATAAGAGAAATTGAAGAGCGCAACGAACTACGAGCATGGCTGGAGGAAAGATTCCTATTGTAGGAATTGTGGCATGTTTACAGCCGGAGATGGGGATAGGATTTCGTGGAGGTCTACCATGGAGGTTGCCCAGTG
AAATGAAGTATATCAGACAGGTCACTTCATTGACGAAAGATCCAAACAAAAAAAATGCTTTGATAATGGGAAGGAAGACATGGGAATCCATACCGCCCAAGTTTCGCCCACTGCCCAATAGAATGAATGTCATTATATCGAGAAGCTTCAAGGAC
GATTTTGTCCACGATAAAGAGAGATCAATAGTCCAAAGTAATTCATTGGCAAACGCAATAATGAACCTAGAAAGCAATTTTAAGGAGCATCTGGAAAGAATCTACGTGATTGGGGGTGGCGAAGTTTATAGTCAAATCTTCTCCATTACAGATCA
TTGGCTCATCACGAAAATAAATCCATTAGATAAAAACGCAACTCCTGCAATGGACACTTTCCTTGATGCGAAGAAATTGGAAGAAGTATTTAGCGAGCAAGATCCGGCCCAGCTGAAAGAATTTCTTCCCCCTAAAGTAGAGTTGCCCGAAACAG
ACTGTGATCAACGCTACTCGCTGGAAGAAAAAGGTTATTGCTTCGAATTCACTCTATACAATCGTAAATGAAACCTCTCCGCCCG

> DFR1 (F38Y)
ACATTATGCTTTGCATGATAATAAGAGAAATTGAAGAGCGCAACGAACTACGAGCATGGCTGGAGGAAAGATTCCTATTGTAGGAATTGTGGCATGTTTACAGCCGGAGATGGGGATAGGATTTCGTGGAGGTCTACCATGGAGGTTGCCCAGTG
AAATGAAGTATTACAGACAGGTCACTTCATTGACGAAAGATCCAAACAAAAAAAATGCTTTGATAATGGGAAGGAAGACATGGGAATCCATACCGCCCAAGTTTCGCCCACTGCCCAATAGAATGAATGTCATTATATCGAGAAGCTTCAAGGAC
GATTTTGTCCACGATAAAGAGAGATCAATAGTCCAAAGTAATTCATTGGCAAACGCAATAATGAACCTAGAAAGCAATTTTAAGGAGCATCTGGAAAGAATCTACGTGATTGGGGGTGGCGAAGTTTATAGTCAAATCTTCTCCATTACAGATCA
TTGGCTCATCACGAAAATAAATCCATTAGATAAAAACGCAACTCCTGCAATGGACACTTTCCTTGATGCGAAGAAATTGGAAGAAGTATTTAGCGAGCAAGATCCGGCCCAGCTGAAAGAATTTCTTCCCCCTAAAGTAGAGTTGCCCGAAACAG
ACTGTGATCAACGCTACTCGCTGGAAGAAAAAGGTTATTGCTTCGAATTCACTCTATACAATCGTAAATGAAACCTCTCCGCCCG

> DFR1 (R39G)
ACATTATGCTTTGCATGATAATAAGAGAAATTGAAGAGCGCAACGAACTACGAGCATGGCTGGAGGAAAGATTCCTATTGTAGGAATTGTGGCATGTTTACAGCCGGAGATGGGGATAGGATTTCGTGGAGGTCTACCATGGAGGTTGCCCAGTG
AAATGAAGTATTTCGGACAGGTCACTTCATTGACGAAAGATCCAAACAAAAAAAATGCTTTGATAATGGGAAGGAAGACATGGGAATCCATACCGCCCAAGTTTCGCCCACTGCCCAATAGAATGAATGTCATTATATCGAGAAGCTTCAAGGAC
GATTTTGTCCACGATAAAGAGAGATCAATAGTCCAAAGTAATTCATTGGCAAACGCAATAATGAACCTAGAAAGCAATTTTAAGGAGCATCTGGAAAGAATCTACGTGATTGGGGGTGGCGAAGTTTATAGTCAAATCTTCTCCATTACAGATCA
TTGGCTCATCACGAAAATAAATCCATTAGATAAAAACGCAACTCCTGCAATGGACACTTTCCTTGATGCGAAGAAATTGGAAGAAGTATTTAGCGAGCAAGATCCGGCCCAGCTGAAAGAATTTCTTCCCCCTAAAGTAGAGTTGCCCGAAACAG
ACTGTGATCAACGCTACTCGCTGGAAGAAAAAGGTTATTGCTTCGAATTCACTCTATACAATCGTAAATGAAACCTCTCCGCCCG

> DFR1 (Q40P)
ACATTATGCTTTGCATGATAATAAGAGAAATTGAAGAGCGCAACGAACTACGAGCATGGCTGGAGGAAAGATTCCTATTGTAGGAATTGTGGCATGTTTACAGCCGGAGATGGGGATAGGATTTCGTGGAGGTCTACCATGGAGGTTGCCCAGTG
AAATGAAGTATTTCAGACCGGTCACTTCATTGACGAAAGATCCAAACAAAAAAAATGCTTTGATAATGGGAAGGAAGACATGGGAATCCATACCGCCCAAGTTTCGCCCACTGCCCAATAGAATGAATGTCATTATATCGAGAAGCTTCAAGGAC
GATTTTGTCCACGATAAAGAGAGATCAATAGTCCAAAGTAATTCATTGGCAAACGCAATAATGAACCTAGAAAGCAATTTTAAGGAGCATCTGGAAAGAATCTACGTGATTGGGGGTGGCGAAGTTTATAGTCAAATCTTCTCCATTACAGATCA
TTGGCTCATCACGAAAATAAATCCATTAGATAAAAACGCAACTCCTGCAATGGACACTTTCCTTGATGCGAAGAAATTGGAAGAAGTATTTAGCGAGCAAGATCCGGCCCAGCTGAAAGAATTTCTTCCCCCTAAAGTAGAGTTGCCCGAAACAG
ACTGTGATCAACGCTACTCGCTGGAAGAAAAAGGTTATTGCTTCGAATTCACTCTATACAATCGTAAATGAAACCTCTCCGCCCG

> DFR1 (I55M)
ACATTATGCTTTGCATGATAATAAGAGAAATTGAAGAGCGCAACGAACTACGAGCATGGCTGGAGGAAAGATTCCTATTGTAGGAATTGTGGCATGTTTACAGCCGGAGATGGGGATAGGATTTCGTGGAGGTCTACCATGGAGGTTGCCCAGTG
AAATGAAGTATTTCAGACAGGTCACTTCATTGACGAAAGATCCAAACAAAAAAAATGCTTTGATGATGGGAAGGAAGACATGGGAATCCATACCGCCCAAGTTTCGCCCACTGCCCAATAGAATGAATGTCATTATATCGAGAAGCTTCAAGGAC
GATTTTGTCCACGATAAAGAGAGATCAATAGTCCAAAGTAATTCATTGGCAAACGCAATAATGAACCTAGAAAGCAATTTTAAGGAGCATCTGGAAAGAATCTACGTGATTGGGGGTGGCGAAGTTTATAGTCAAATCTTCTCCATTACAGATCA
TTGGCTCATCACGAAAATAAATCCATTAGATAAAAACGCAACTCCTGCAATGGACACTTTCCTTGATGCGAAGAAATTGGAAGAAGTATTTAGCGAGCAAGATCCGGCCCAGCTGAAAGAATTTCTTCCCCCTAAAGTAGAGTTGCCCGAAACAG
ACTGTGATCAACGCTACTCGCTGGAAGAAAAAGGTTATTGCTTCGAATTCACTCTATACAATCGTAAATGAAACCTCTCCGCCCG

> DFR1 (S63A)
ACATTATGCTTTGCATGATAATAAGAGAAATTGAAGAGCGCAACGAACTACGAGCATGGCTGGAGGAAAGATTCCTATTGTAGGAATTGTGGCATGTTTACAGCCGGAGATGGGGATAGGATTTCGTGGAGGTCTACCATGGAGGTTGCCCAGTG
AAATGAAGTATTTCAGACAGGTCACTTCATTGACGAAAGATCCAAACAAAAAAAATGCTTTGATAATGGGAAGGAAGACATGGGAAGCCATACCGCCCAAGTTTCGCCCACTGCCCAATAGAATGAATGTCATTATATCGAGAAGCTTCAAGGAC
GATTTTGTCCACGATAAAGAGAGATCAATAGTCCAAAGTAATTCATTGGCAAACGCAATAATGAACCTAGAAAGCAATTTTAAGGAGCATCTGGAAAGAATCTACGTGATTGGGGGTGGCGAAGTTTATAGTCAAATCTTCTCCATTACAGATCA
TTGGCTCATCACGAAAATAAATCCATTAGATAAAAACGCAACTCCTGCAATGGACACTTTCCTTGATGCGAAGAAATTGGAAGAAGTATTTAGCGAGCAAGATCCGGCCCAGCTGAAAGAATTTCTTCCCCCTAAAGTAGAGTTGCCCGAAACAG
ACTGTGATCAACGCTACTCGCTGGAAGAAAAAGGTTATTGCTTCGAATTCACTCTATACAATCGTAAATGAAACCTCTCCGCCCG

> DFR1 (I64T)
ACATTATGCTTTGCATGATAATAAGAGAAATTGAAGAGCGCAACGAACTACGAGCATGGCTGGAGGAAAGATTCCTATTGTAGGAATTGTGGCATGTTTACAGCCGGAGATGGGGATAGGATTTCGTGGAGGTCTACCATGGAGGTTGCCCAGTG
AAATGAAGTATTTCAGACAGGTCACTTCATTGACGAAAGATCCAAACAAAAAAAATGCTTTGATAATGGGAAGGAAGACATGGGAATCCACACCGCCCAAGTTTCGCCCACTGCCCAATAGAATGAATGTCATTATATCGAGAAGCTTCAAGGAC
GATTTTGTCCACGATAAAGAGAGATCAATAGTCCAAAGTAATTCATTGGCAAACGCAATAATGAACCTAGAAAGCAATTTTAAGGAGCATCTGGAAAGAATCTACGTGATTGGGGGTGGCGAAGTTTATAGTCAAATCTTCTCCATTACAGATCA
TTGGCTCATCACGAAAATAAATCCATTAGATAAAAACGCAACTCCTGCAATGGACACTTTCCTTGATGCGAAGAAATTGGAAGAAGTATTTAGCGAGCAAGATCCGGCCCAGCTGAAAGAATTTCTTCCCCCTAAAGTAGAGTTGCCCGAAACAG
ACTGTGATCAACGCTACTCGCTGGAAGAAAAAGGTTATTGCTTCGAATTCACTCTATACAATCGTAAATGAAACCTCTCCGCCCG

> DFR1 (I64M)

ACATTATGCTTTGCATGATAATAAGAGAAATTGAAGAGCGCAACGAACTACGAGCATGGCTGGAGGAAAGATTCCTATTGTAGGAATTGTGGCATGTTTACAGCCGGAGATGGGGATAGGATTTCGTGGAGGTCTACCATGGAGGTTGCCCAGTG
AAATGAAGTATTTCAGACAGGTCACTTCATTGACGAAAGATCCAAACAAAAAAAATGCTTTGATAATGGGAAGGAAGACATGGGAATCCATGCCGCCCAAGTTTCGCCCACTGCCCAATAGAATGAATGTCATTATATCGAGAAGCTTCAAGGAC
GATTTTGTCCACGATAAAGAGAGATCAATAGTCCAAAGTAATTCATTGGCAAACGCAATAATGAACCTAGAAAGCAATTTTAAGGAGCATCTGGAAAGAATCTACGTGATTGGGGGTGGCGAAGTTTATAGTCAAATCTTCTCCATTACAGATCA
TTGGCTCATCACGAAAATAAATCCATTAGATAAAAACGCAACTCCTGCAATGGACACTTTCCTTGATGCGAAGAAATTGGAAGAAGTATTTAGCGAGCAAGATCCGGCCCAGCTGAAAGAATTTCTTCCCCCTAAAGTAGAGTTGCCCGAAACAG
ACTGTGATCAACGCTACTCGCTGGAAGAAAAAGGTTATTGCTTCGAATTCACTCTATACAATCGTAAATGAAACCTCTCCGCCCG

> DFR1 (P65P)
ACATTATGCTTTGCATGATAATAAGAGAAATTGAAGAGCGCAACGAACTACGAGCATGGCTGGAGGAAAGATTCCTATTGTAGGAATTGTGGCATGTTTACAGCCGGAGATGGGGATAGGATTTCGTGGAGGTCTACCATGGAGGTTGCCCAGTG
AAATGAAGTATTTCAGACAGGTCACTTCATTGACGAAAGATCCAAACAAAAAAAATGCTTTGATAATGGGAAGGAAGACATGGGAATCCATACCACCCAAGTTTCGCCCACTGCCCAATAGAATGAATGTCATTATATCGAGAAGCTTCAAGGAC
GATTTTGTCCACGATAAAGAGAGATCAATAGTCCAAAGTAATTCATTGGCAAACGCAATAATGAACCTAGAAAGCAATTTTAAGGAGCATCTGGAAAGAATCTACGTGATTGGGGGTGGCGAAGTTTATAGTCAAATCTTCTCCATTACAGATCA
TTGGCTCATCACGAAAATAAATCCATTAGATAAAAACGCAACTCCTGCAATGGACACTTTCCTTGATGCGAAGAAATTGGAAGAAGTATTTAGCGAGCAAGATCCGGCCCAGCTGAAAGAATTTCTTCCCCCTAAAGTAGAGTTGCCCGAAACAG
ACTGTGATCAACGCTACTCGCTGGAAGAAAAAGGTTATTGCTTCGAATTCACTCTATACAATCGTAAATGAAACCTCTCCGCCCG

> DFR1 (F68L)
ACATTATGCTTTGCATGATAATAAGAGAAATTGAAGAGCGCAACGAACTACGAGCATGGCTGGAGGAAAGATTCCTATTGTAGGAATTGTGGCATGTTTACAGCCGGAGATGGGGATAGGATTTCGTGGAGGTCTACCATGGAGGTTGCCCAGTG
AAATGAAGTATTTCAGACAGGTCACTTCATTGACGAAAGATCCAAACAAAAAAAATGCTTTGATAATGGGAAGGAAGACATGGGAATCCATACCGCCCAAGCTTCGCCCACTGCCCAATAGAATGAATGTCATTATATCGAGAAGCTTCAAGGAC
GATTTTGTCCACGATAAAGAGAGATCAATAGTCCAAAGTAATTCATTGGCAAACGCAATAATGAACCTAGAAAGCAATTTTAAGGAGCATCTGGAAAGAATCTACGTGATTGGGGGTGGCGAAGTTTATAGTCAAATCTTCTCCATTACAGATCA
TTGGCTCATCACGAAAATAAATCCATTAGATAAAAACGCAACTCCTGCAATGGACACTTTCCTTGATGCGAAGAAATTGGAAGAAGTATTTAGCGAGCAAGATCCGGCCCAGCTGAAAGAATTTCTTCCCCCTAAAGTAGAGTTGCCCGAAACAG
ACTGTGATCAACGCTACTCGCTGGAAGAAAAAGGTTATTGCTTCGAATTCACTCTATACAATCGTAAATGAAACCTCTCCGCCCG

> DFR1 (E109K)
ACATTATGCTTTGCATGATAATAAGAGAAATTGAAGAGCGCAACGAACTACGAGCATGGCTGGAGGAAAGATTCCTATTGTAGGAATTGTGGCATGTTTACAGCCGGAGATGGGGATAGGATTTCGTGGAGGTCTACCATGGAGGTTGCCCAGTG
AAATGAAGTATTTCAGACAGGTCACTTCATTGACGAAAGATCCAAACAAAAAAAATGCTTTGATAATGGGAAGGAAGACATGGGAATCCATACCGCCCAAGTTTCGCCCACTGCCCAATAGAATGAATGTCATTATATCGAGAAGCTTCAAGGAC
GATTTTGTCCACGATAAAGAGAGATCAATAGTCCAAAGTAATTCATTGGCAAACGCAATAATGAACCTAAAAAGCAATTTTAAGGAGCATCTGGAAAGAATCTACGTGATTGGGGGTGGCGAAGTTTATAGTCAAATCTTCTCCATTACAGATCA
TTGGCTCATCACGAAAATAAATCCATTAGATAAAAACGCAACTCCTGCAATGGACACTTTCCTTGATGCGAAGAAATTGGAAGAAGTATTTAGCGAGCAAGATCCGGCCCAGCTGAAAGAATTTCTTCCCCCTAAAGTAGAGTTGCCCGAAACAG
ACTGTGATCAACGCTACTCGCTGGAAGAAAAAGGTTATTGCTTCGAATTCACTCTATACAATCGTAAATGAAACCTCTCCGCCCG

> DFR1 (R118G)
ACATTATGCTTTGCATGATAATAAGAGAAATTGAAGAGCGCAACGAACTACGAGCATGGCTGGAGGAAAGATTCCTATTGTAGGAATTGTGGCATGTTTACAGCCGGAGATGGGGATAGGATTTCGTGGAGGTCTACCATGGAGGTTGCCCAGTG
AAATGAAGTATTTCAGACAGGTCACTTCATTGACGAAAGATCCAAACAAAAAAAATGCTTTGATAATGGGAAGGAAGACATGGGAATCCATACCGCCCAAGTTTCGCCCACTGCCCAATAGAATGAATGTCATTATATCGAGAAGCTTCAAGGAC
GATTTTGTCCACGATAAAGAGAGATCAATAGTCCAAAGTAATTCATTGGCAAACGCAATAATGAACCTAGAAAGCAATTTTAAGGAGCATCTGGAAGGAATCTACGTGATTGGGGGTGGCGAAGTTTATAGTCAAATCTTCTCCATTACAGATCA
TTGGCTCATCACGAAAATAAATCCATTAGATAAAAACGCAACTCCTGCAATGGACACTTTCCTTGATGCGAAGAAATTGGAAGAAGTATTTAGCGAGCAAGATCCGGCCCAGCTGAAAGAATTTCTTCCCCCTAAAGTAGAGTTGCCCGAAACAG
ACTGTGATCAACGCTACTCGCTGGAAGAAAAAGGTTATTGCTTCGAATTCACTCTATACAATCGTAAATGAAACCTCTCCGCCCG

> DFR1 (T141A)
ACATTATGCTTTGCATGATAATAAGAGAAATTGAAGAGCGCAACGAACTACGAGCATGGCTGGAGGAAAGATTCCTATTGTAGGAATTGTGGCATGTTTACAGCCGGAGATGGGGATAGGATTTCGTGGAGGTCTACCATGGAGGTTGCCCAGTG
AAATGAAGTATTTCAGACAGGTCACTTCATTGACGAAAGATCCAAACAAAAAAAATGCTTTGATAATGGGAAGGAAGACATGGGAATCCATACCGCCCAAGTTTCGCCCACTGCCCAATAGAATGAATGTCATTATATCGAGAAGCTTCAAGGAC
GATTTTGTCCACGATAAAGAGAGATCAATAGTCCAAAGTAATTCATTGGCAAACGCAATAATGAACCTAGAAAGCAATTTTAAGGAGCATCTGGAAAGAATCTACGTGATTGGGGGTGGCGAAGTTTATAGTCAAATCTTCTCCATTACAGATCA
TTGGCTCATCGCGAAAATAAATCCATTAGATAAAAACGCAACTCCTGCAATGGACACTTTCCTTGATGCGAAGAAATTGGAAGAAGTATTTAGCGAGCAAGATCCGGCCCAGCTGAAAGAATTTCTTCCCCCTAAAGTAGAGTTGCCCGAAACAG
ACTGTGATCAACGCTACTCGCTGGAAGAAAAAGGTTATTGCTTCGAATTCACTCTATACAATCGTAAATGAAACCTCTCCGCCCG

> DFR1 (D155H)
ACATTATGCTTTGCATGATAATAAGAGAAATTGAAGAGCGCAACGAACTACGAGCATGGCTGGAGGAAAGATTCCTATTGTAGGAATTGTGGCATGTTTACAGCCGGAGATGGGGATAGGATTTCGTGGAGGTCTACCATGGAGGTTGCCCAGTG
AAATGAAGTATTTCAGACAGGTCACTTCATTGACGAAAGATCCAAACAAAAAAAATGCTTTGATAATGGGAAGGAAGACATGGGAATCCATACCGCCCAAGTTTCGCCCACTGCCCAATAGAATGAATGTCATTATATCGAGAAGCTTCAAGGAC
GATTTTGTCCACGATAAAGAGAGATCAATAGTCCAAAGTAATTCATTGGCAAACGCAATAATGAACCTAGAAAGCAATTTTAAGGAGCATCTGGAAAGAATCTACGTGATTGGGGGTGGCGAAGTTTATAGTCAAATCTTCTCCATTACAGATCA
TTGGCTCATCACGAAAATAAATCCATTAGATAAAAACGCAACTCCTGCAATGCACACTTTCCTTGATGCGAAGAAATTGGAAGAAGTATTTAGCGAGCAAGATCCGGCCCAGCTGAAAGAATTTCTTCCCCCTAAAGTAGAGTTGCCCGAAACAG
ACTGTGATCAACGCTACTCGCTGGAAGAAAAAGGTTATTGCTTCGAATTCACTCTATACAATCGTAAATGAAACCTCTCCGCCCG

> DFR1 (T156A)
ACATTATGCTTTGCATGATAATAAGAGAAATTGAAGAGCGCAACGAACTACGAGCATGGCTGGAGGAAAGATTCCTATTGTAGGAATTGTGGCATGTTTACAGCCGGAGATGGGGATAGGATTTCGTGGAGGTCTACCATGGAGGTTGCCCAGTG
AAATGAAGTATTTCAGACAGGTCACTTCATTGACGAAAGATCCAAACAAAAAAAATGCTTTGATAATGGGAAGGAAGACATGGGAATCCATACCGCCCAAGTTTCGCCCACTGCCCAATAGAATGAATGTCATTATATCGAGAAGCTTCAAGGAC
GATTTTGTCCACGATAAAGAGAGATCAATAGTCCAAAGTAATTCATTGGCAAACGCAATAATGAACCTAGAAAGCAATTTTAAGGAGCATCTGGAAAGAATCTACGTGATTGGGGGTGGCGAAGTTTATAGTCAAATCTTCTCCATTACAGATCA
TTGGCTCATCACGAAAATAAATCCATTAGATAAAAACGCAACTCCTGCAATGGACGCTTTCCTTGATGCGAAGAAATTGGAAGAAGTATTTAGCGAGCAAGATCCGGCCCAGCTGAAAGAATTTCTTCCCCCTAAAGTAGAGTTGCCCGAAACAG
ACTGTGATCAACGCTACTCGCTGGAAGAAAAAGGTTATTGCTTCGAATTCACTCTATACAATCGTAAATGAAACCTCTCCGCCCG

> DFR1 (F167L)
ACATTATGCTTTGCATGATAATAAGAGAAATTGAAGAGCGCAACGAACTACGAGCATGGCTGGAGGAAAGATTCCTATTGTAGGAATTGTGGCATGTTTACAGCCGGAGATGGGGATAGGATTTCGTGGAGGTCTACCATGGAGGTTGCCCAGTG
AAATGAAGTATTTCAGACAGGTCACTTCATTGACGAAAGATCCAAACAAAAAAAATGCTTTGATAATGGGAAGGAAGACATGGGAATCCATACCGCCCAAGTTTCGCCCACTGCCCAATAGAATGAATGTCATTATATCGAGAAGCTTCAAGGAC
GATTTTGTCCACGATAAAGAGAGATCAATAGTCCAAAGTAATTCATTGGCAAACGCAATAATGAACCTAGAAAGCAATTTTAAGGAGCATCTGGAAAGAATCTACGTGATTGGGGGTGGCGAAGTTTATAGTCAAATCTTCTCCATTACAGATCA
TTGGCTCATCACGAAAATAAATCCATTAGATAAAAACGCAACTCCTGCAATGGACACTTTCCTTGATGCGAAGAAATTGGAAGAAGTATTGAGCGAGCAAGATCCGGCCCAGCTGAAAGAATTTCTTCCCCCTAAAGTAGAGTTGCCCGAAACAG
ACTGTGATCAACGCTACTCGCTGGAAGAAAAAGGTTATTGCTTCGAATTCACTCTATACAATCGTAAATGAAACCTCTCCGCCCG

> DFR1 (K176T)
ACATTATGCTTTGCATGATAATAAGAGAAATTGAAGAGCGCAACGAACTACGAGCATGGCTGGAGGAAAGATTCCTATTGTAGGAATTGTGGCATGTTTACAGCCGGAGATGGGGATAGGATTTCGTGGAGGTCTACCATGGAGGTTGCCCAGTG
AAATGAAGTATTTCAGACAGGTCACTTCATTGACGAAAGATCCAAACAAAAAAAATGCTTTGATAATGGGAAGGAAGACATGGGAATCCATACCGCCCAAGTTTCGCCCACTGCCCAATAGAATGAATGTCATTATATCGAGAAGCTTCAAGGAC
GATTTTGTCCACGATAAAGAGAGATCAATAGTCCAAAGTAATTCATTGGCAAACGCAATAATGAACCTAGAAAGCAATTTTAAGGAGCATCTGGAAAGAATCTACGTGATTGGGGGTGGCGAAGTTTATAGTCAAATCTTCTCCATTACAGATCA
TTGGCTCATCACGAAAATAAATCCATTAGATAAAAACGCAACTCCTGCAATGGACACTTTCCTTGATGCGAAGAAATTGGAAGAAGTATTTAGCGAGCAAGATCCGGCCCAGCTGACAGAATTTCTTCCCCCTAAAGTAGAGTTGCCCGAAACAG
ACTGTGATCAACGCTACTCGCTGGAAGAAAAAGGTTATTGCTTCGAATTCACTCTATACAATCGTAAATGAAACCTCTCCGCCCG

> DFR1 (F178F)
ACATTATGCTTTGCATGATAATAAGAGAAATTGAAGAGCGCAACGAACTACGAGCATGGCTGGAGGAAAGATTCCTATTGTAGGAATTGTGGCATGTTTACAGCCGGAGATGGGGATAGGATTTCGTGGAGGTCTACCATGGAGGTTGCCCAGTG
AAATGAAGTATTTCAGACAGGTCACTTCATTGACGAAAGATCCAAACAAAAAAAATGCTTTGATAATGGGAAGGAAGACATGGGAATCCATACCGCCCAAGTTTCGCCCACTGCCCAATAGAATGAATGTCATTATATCGAGAAGCTTCAAGGAC
GATTTTGTCCACGATAAAGAGAGATCAATAGTCCAAAGTAATTCATTGGCAAACGCAATAATGAACCTAGAAAGCAATTTTAAGGAGCATCTGGAAAGAATCTACGTGATTGGGGGTGGCGAAGTTTATAGTCAAATCTTCTCCATTACAGATCA
TTGGCTCATCACGAAAATAAATCCATTAGATAAAAACGCAACTCCTGCAATGGACACTTTCCTTGATGCGAAGAAATTGGAAGAAGTATTTAGCGAGCAAGATCCGGCCCAGCTGAAAGAATTCCTTCCCCCTAAAGTAGAGTTGCCCGAAACAG
ACTGTGATCAACGCTACTCGCTGGAAGAAAAAGGTTATTGCTTCGAATTCACTCTATACAATCGTAAATGAAACCTCTCCGCCCG

> DFR1 (E187K)
ACATTATGCTTTGCATGATAATAAGAGAAATTGAAGAGCGCAACGAACTACGAGCATGGCTGGAGGAAAGATTCCTATTGTAGGAATTGTGGCATGTTTACAGCCGGAGATGGGGATAGGATTTCGTGGAGGTCTACCATGGAGGTTGCCCAGTG
AAATGAAGTATTTCAGACAGGTCACTTCATTGACGAAAGATCCAAACAAAAAAAATGCTTTGATAATGGGAAGGAAGACATGGGAATCCATACCGCCCAAGTTTCGCCCACTGCCCAATAGAATGAATGTCATTATATCGAGAAGCTTCAAGGAC
GATTTTGTCCACGATAAAGAGAGATCAATAGTCCAAAGTAATTCATTGGCAAACGCAATAATGAACCTAGAAAGCAATTTTAAGGAGCATCTGGAAAGAATCTACGTGATTGGGGGTGGCGAAGTTTATAGTCAAATCTTCTCCATTACAGATCA
TTGGCTCATCACGAAAATAAATCCATTAGATAAAAACGCAACTCCTGCAATGGACACTTTCCTTGATGCGAAGAAATTGGAAGAAGTATTTAGCGAGCAAGATCCGGCCCAGCTGAAAGAATTTCTTCCCCCTAAAGTAGAGTTGCCCAAAACAG
ACTGTGATCAACGCTACTCGCTGGAAGAAAAAGGTTATTGCTTCGAATTCACTCTATACAATCGTAAATGAAACCTCTCCGCCCG

> DFR1 (K199E)
ACATTATGCTTTGCATGATAATAAGAGAAATTGAAGAGCGCAACGAACTACGAGCATGGCTGGAGGAAAGATTCCTATTGTAGGAATTGTGGCATGTTTACAGCCGGAGATGGGGATAGGATTTCGTGGAGGTCTACCATGGAGGTTGCCCAGTG
AAATGAAGTATTTCAGACAGGTCACTTCATTGACGAAAGATCCAAACAAAAAAAATGCTTTGATAATGGGAAGGAAGACATGGGAATCCATACCGCCCAAGTTTCGCCCACTGCCCAATAGAATGAATGTCATTATATCGAGAAGCTTCAAGGAC
GATTTTGTCCACGATAAAGAGAGATCAATAGTCCAAAGTAATTCATTGGCAAACGCAATAATGAACCTAGAAAGCAATTTTAAGGAGCATCTGGAAAGAATCTACGTGATTGGGGGTGGCGAAGTTTATAGTCAAATCTTCTCCATTACAGATCA
TTGGCTCATCACGAAAATAAATCCATTAGATAAAAACGCAACTCCTGCAATGGACACTTTCCTTGATGCGAAGAAATTGGAAGAAGTATTTAGCGAGCAAGATCCGGCCCAGCTGAAAGAATTTCTTCCCCCTAAAGTAGAGTTGCCCGAAACAG
ACTGTGATCAACGCTACTCGCTGGAAGAAGAAGGTTATTGCTTCGAATTCACTCTATACAATCGTAAATGAAACCTCTCCGCCCG

> DFR1 (C202F)
ACATTATGCTTTGCATGATAATAAGAGAAATTGAAGAGCGCAACGAACTACGAGCATGGCTGGAGGAAAGATTCCTATTGTAGGAATTGTGGCATGTTTACAGCCGGAGATGGGGATAGGATTTCGTGGAGGTCTACCATGGAGGTTGCCCAGTG
AAATGAAGTATTTCAGACAGGTCACTTCATTGACGAAAGATCCAAACAAAAAAAATGCTTTGATAATGGGAAGGAAGACATGGGAATCCATACCGCCCAAGTTTCGCCCACTGCCCAATAGAATGAATGTCATTATATCGAGAAGCTTCAAGGAC
GATTTTGTCCACGATAAAGAGAGATCAATAGTCCAAAGTAATTCATTGGCAAACGCAATAATGAACCTAGAAAGCAATTTTAAGGAGCATCTGGAAAGAATCTACGTGATTGGGGGTGGCGAAGTTTATAGTCAAATCTTCTCCATTACAGATCA
TTGGCTCATCACGAAAATAAATCCATTAGATAAAAACGCAACTCCTGCAATGGACACTTTCCTTGATGCGAAGAAATTGGAAGAAGTATTTAGCGAGCAAGATCCGGCCCAGCTGAAAGAATTTCTTCCCCCTAAAGTAGAGTTGCCCGAAACAG
ACTGTGATCAACGCTACTCGCTGGAAGAAAAAGGTTATTTCTTCGAATTCACTCTATACAATCGTAAATGAAACCTCTCCGCCCG

> DFR1 (N209H)
ACATTATGCTTTGCATGATAATAAGAGAAATTGAAGAGCGCAACGAACTACGAGCATGGCTGGAGGAAAGATTCCTATTGTAGGAATTGTGGCATGTTTACAGCCGGAGATGGGGATAGGATTTCGTGGAGGTCTACCATGGAGGTTGCCCAGTG
AAATGAAGTATTTCAGACAGGTCACTTCATTGACGAAAGATCCAAACAAAAAAAATGCTTTGATAATGGGAAGGAAGACATGGGAATCCATACCGCCCAAGTTTCGCCCACTGCCCAATAGAATGAATGTCATTATATCGAGAAGCTTCAAGGAC
GATTTTGTCCACGATAAAGAGAGATCAATAGTCCAAAGTAATTCATTGGCAAACGCAATAATGAACCTAGAAAGCAATTTTAAGGAGCATCTGGAAAGAATCTACGTGATTGGGGGTGGCGAAGTTTATAGTCAAATCTTCTCCATTACAGATCA
TTGGCTCATCACGAAAATAAATCCATTAGATAAAAACGCAACTCCTGCAATGGACACTTTCCTTGATGCGAAGAAATTGGAAGAAGTATTTAGCGAGCAAGATCCGGCCCAGCTGAAAGAATTTCTTCCCCCTAAAGTAGAGTTGCCCGAAACAG
ACTGTGATCAACGCTACTCGCTGGAAGAAAAAGGTTATTGCTTCGAATTCACTCTATACCATCGTAAATGAAACCTCTCCGCCCG
